# Miltefosine reinvigorates exhausted T cells by targeting their bioenergetic state

**DOI:** 10.1016/j.xcrm.2024.101869

**Published:** 2024-12-09

**Authors:** Xingying Zhang, Chenze Zhang, Shan Lu, Jingxi Dong, Na Tang, Yao Wang, Weidong Han, Xi Pan, Xiang Zhang, Duan Liu, Ng Shyh-Chang, Yu Wang, Guihai Feng, Haoyi Wang

**Affiliations:** 1 State Key Laboratory of Stem Cell and Reproductive Biology, Institute of Zoology, Chinese Academy of Sciences, Beijing 100101, China; 2 National Key Laboratory of Efficacy and Mechanism on Chinese Medicine for Metabolic Diseases, Beijing Research Institute of Chinese Medicine, Beijing University of Chinese Medicine, Beijing 102488, China; 3 University of Chinese Academy of Sciences, Beijing 100049, China; 4 Beijing Institute for Stem Cell and Regenerative Medicine, Beijing 100101, China; 5 Chinese People’s Liberation Army General Hospital, Beijing 100176, China; 6 Institute for Stem Cell and Regeneration, Chinese Academy of Sciences, Beijing 100101, China; 7 College of Life Sciences and Oceanography, Shenzhen University, Shenzhen 518060, China

**Keywords:** T cell exhaustion, high-throughput drug screening, miltefosine, glycolytic metabolism, immunotherapy for solid tumors

## Abstract

T cell exhaustion presents a major challenge for the efficacy of both immune checkpoint inhibitors (ICBs) and chimeric antigen receptor T (CAR-T) cell immunotherapies. To address this issue, we generate hypofunctional CAR-T cells that imitate the exhaustion state. By screening a Food and Drug Administration (FDA)-approved small molecule library using this model, we identify miltefosine as a potent molecule that restores the impaired function of CAR-T cells in a PD-1/PD-L1-independent manner. Impressively, in the terminally exhausted state where PD-1 antibody treatment is ineffective, miltefosine still enhances CAR-T cell activity. Single-cell sequencing analysis reveals that miltefosine treatment significantly increases the population of effector cells. Mechanistically, miltefosine improves impaired glycolysis and oxidative phosphorylation in hypofunctional CAR-T cells. In both allogeneic and syngeneic tumor models, miltefosine effectively enhances the solid tumor clearance ability of CAR-T cells and T cells, demonstrating its potential as an effective immunotherapeutic drug.

## Highlights

• Hypofunctional CAR-T cells generated via repeated tumor challenge
• Miltefosine is identified as an enhancer of hypofunctional CAR-T cells
• Miltefosine restores glycolytic metabolism and glucose uptake in CAR-T cells
• Miltefosine improves CAR-T and T cell efficacy against solid tumors *in vivo*

## Graphical abstract

**Figure:**
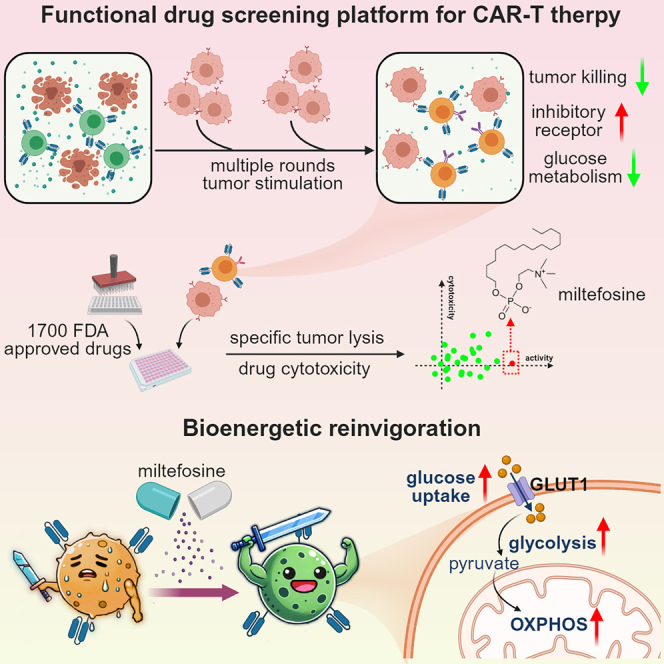

## Teaser

Zhang et al. establish a drug screening platform using a CAR-T cell exhaustion model to evaluate FDA-approved small molecules. Miltefosine emerges as a promising candidate, demonstrating the ability to restore CAR-T cell function and enhance metabolic activity, highlighting its potential as an effective immunotherapy.

## Introduction

T cell exhaustion is a differentiation state acquired when T cells are exposed to persistent antigen stimulation,^1^^,^^2^ characterized by progressive loss of effector functions, stable expression of inhibitory receptors, distinct epigenetic profiles, defective cytokine production, impaired proliferation capacity, and suppressed mitochondrial respiration and glycolysis function.^3^^,^^4^ Originally identified in the chronic lymphocytic choriomeningitis virus (LCMV) infected mouse model,^5^^,^^6^^,^^7^ T cell exhaustion is now appreciated to occur in diverse diseases, including malignant tumors.^8^^,^^9^^,^^10^ Exhausted CD8^+^ T cells are phenotypically and functionally heterogeneous. Recently, two subtypes of exhausted CD8^+^ tumor-infiltrating lymphocytes (TILs) were identified, each with distinct functional properties.^11^^,^^12^^,^^13^^,^^14^ Progenitor-exhausted CD8^+^ T cells exhibit a relatively high proliferative capacity and can respond to anti-PD-1 checkpoint blockade therapy. In contrast, terminally exhausted CD8^+^ TILs have impaired proliferative capacity and do not respond to most existing immunotherapies, including immune checkpoint blockers (ICBs). In addition, single-cell RNA sequencing (scRNA-seq) analysis has been used to characterize the landscapes of TILs in various types of cancer, including liver cancer, colorectal cancer, and non-small cell lung cancer,^15^^,^^16^^,^^17^ providing an invaluable reference for studying exhausted T cells in cancer. Importantly, studies have demonstrated that T cell exhaustion represents a major barrier to the efficacy of both ICB and chimeric antigen receptor T (CAR-T) cell immunotherapies, and manipulating this process may lead to improved efficacy of T cell responses in cancer.^18^^,^^19^

To overcome T cell exhaustion, various strategies have been proposed, including the manipulation of genes involved in T cell exhaustion. *TOX*,^20^^,^^21^^,^^22^^,^^23^^,^^24^ *NR4A*,^22^^,^^25^^,^^26^ *BATF*,^27^^,^^28^ *SNX9*,^29^ *CBLB*,^30^ *TGFBR2*,^31^ *ARID1A*,^32^ and *REGNASE-1* and *ROQUIN-1*^33^^,^^34^^,^^35^ have been reported to induce T cell exhaustion. Knockout of these genes can enhance T cell function. Additionally, c-JUN—an AP-1 family transcription factor^36^—lymphotoxin beta receptor (LTBR),^37^ interleukin (IL)-7, or C-C motif chemokine ligand (CCL)19^38^ have been shown to mitigate CAR-T cell exhaustion, and their overexpression can enhance tumor elimination ability in various studies. However, the clinical efficacy of CAR-T therapy treating solid tumor is still limited. Additionally, although ICB therapies have shown remarkable clinical success in treating cancer patients, only a subset of patients achieved complete remission after treatment.^39^^,^^40^ Therefore, the discovery of effective targets and drugs to overcome the limitations of current treatments is highly desirable.

In this study, we developed a functional screening platform using primary human T cells, to identify compounds that rejuvenate exhausted T cells and improve their efficacy. Using this platform, we conducted a screen of Food and Drug Administration (FDA)-approved drugs and identified that the small molecule miltefosine, previously used as an antiparasitic drug to treat leishmaniasis,^41^ can enhance the efficacy of exhausted CAR-T cells. Further investigation revealed that miltefosine rescued glycolysis and oxidative phosphorylation (OXPHOS) metabolism defects in exhausted T cells, leading to improved efficacy in treating solid tumors.

## Results

### Generating hypofunctional CAR-T cells via multiple rounds of tumor challenge

**Figure 1:**
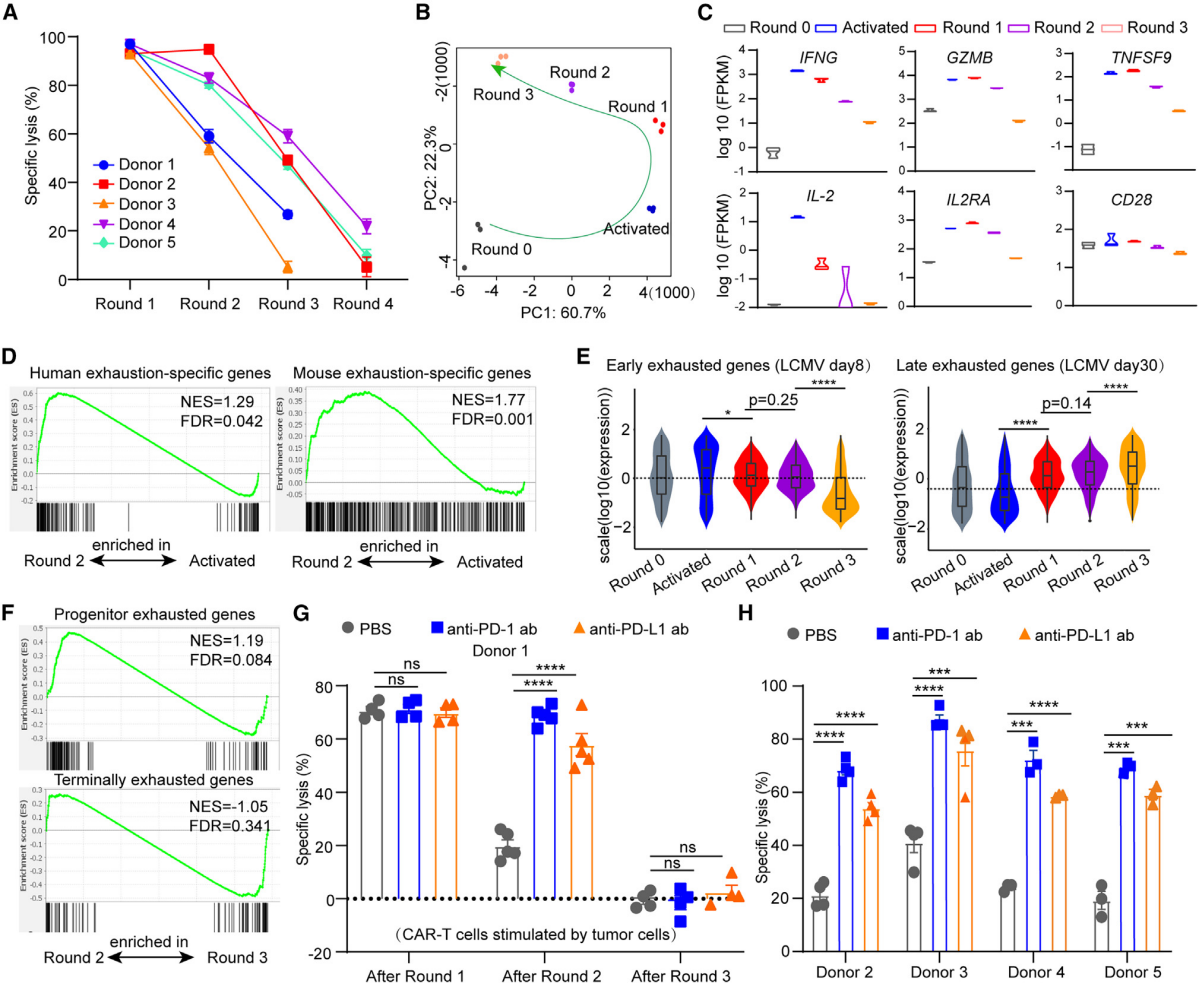
Generating hypofunction CAR-T cells by multiple rounds of tumor challenge and reinvigorating them with anti-PD-1/PD-L1 blockade (A) The specific lysis of NCI-H226-luciferase after coculture with M28Z generated from 4 donors upon multiple rounds of tumor challenge (*n* = 4). (B) The PCA of round 0, activated, round 1, round 2, and round 3 M28Z CAR-T cells. (C) Log_10_ FPKM of activation- and proliferation-related genes in different samples. FPKM, fragments per kilobase of exon model per million mapped fragments. (D) GSEA analysis was performed on genes upregulated in CD8^+^ T cell exhaustion-specific genes in humans (including liver cancer,^15^ colorectal cancer,^17^ and non-small-cell lung cancer^16^ and genes upregulated in T cell exhaustion-specific genes in chronic LCMV infection in mice.^42^^,^^43^ The normalized enrichment score (NES) was used. The genes, rank-ordered from left to right, were enriched in the M28Z (round 2) and M28Z (activated) groups, respectively. (E) The expression of early exhausted makers and late exhausted makers^6^ in different groups. The dashed line represents expression of round 0 CAR-T cells. (F) GSEA of genes upregulated in progenitor and terminally exhaustion-related genes.^12^ Genes from the left to right of the rank order were enriched in the M28Z (round 2) and M28Z (round 3) groups. (G) The specific lysis of NCI-H226-luciferase cells after coculture with round 1, round 2, and round 3 M28Z CAR-T cells (donor 1) with anti-PD-1 or anti-PD-L1 antibody treatment for 4 days at a 1:1 E:T ratio (*n* = 4). (H) The specific lysis of NCI-H226-luciferase after coculture with hypofunctional M28Z (donors 2–5) for 4 days at 1:1 E:T ratio with anti-PD-1 or anti-PD-L1 antibody treatment (*n* = 4, results of other E:T ratios shown in Figure S1H). Unpaired t test was used in statistical analysis. NS, not significant, ∗*p* < 0.05, ∗∗∗*p* < 0.001, ∗∗∗∗*p* < 0.0001. All error bars denote SEM. See also Figure S1.

In our previous work, we generated hypofunctional CAR-T cells by reducing the E:T (effector cell: target cell) ratio and prolonging the coculture time. The CAR-T cells obtained from this model exhibited transcriptomic and functional profiles similar to those of exhausted T cells *in vivo.*^27^ However, the yield of exhausted CAR-T cells using this approach is relatively low. To generate a larger number of exhausted CAR-T cells for high-throughput screening, we developed a protocol that subjects CAR-T cells to multiple rounds of tumor cell challenge, which induces them into a hypofunctional state (Figure S1A). The CAR (M28Z) employed in this study contained a human mesothelin-binding single-chain antibody fragment (scFv), CD28 costimulatory, a CD3-zeta domain, and a GFP reporter linked via P2A.^27^^,^^31^ As shown in Figure 1A, we generated hypofunctional CAR-T cells by subjecting M28Z cells to multiple rounds of stimulation of a mesothelin-positive lung cancer cell line (NCI-H226-luciferase) at a 1:1 E:T ratio. As a result, M28Z cells gradually reduced their tumor-killing capability, while the dynamics varied among different donors (Figure 1A).

To further characterize these exhausted cells, we performed bulk RNA sequencing (RNA-seq) on CD8-positive cells from donor 1 (referred to in Figure 1A) after each round of tumor stimulation. Additionally, we sorted CAR-T cells that had been cocultured with tumor cells for 24 h at an E:T ratio of 2:1 (resulting in 90% tumor lysis) and labeled them as activated M28Z; these served as the activation state control. Principal-component analysis (PCA) highlighted the distinct transcriptional program for CAR-T cells upon different rounds of tumor challenge (Figure 1B). The expression of activation-specific genes (*IFNG*, *GZMB*, and *TNFRSF9*) and cell proliferation-specific genes (*IL2*, *IL2RA*, and *CD28*) decreased with the increasing rounds of tumor stimulation (Figure 1C), especially from round 2 (the round in which tumor-killing ability begins to decline). Gene set enrichment analysis (GSEA) showed that the T cell exhaustion-specific genes, identified from patients^15^^,^^16^^,^^17^ and mice,^7^^,^^42^ were significantly enriched in round 2 M28Z compared with activated M28Z cells (donor 1) (Figure 1D), revealing that the second round stimulated CAR-T cells from this coculture model displayed a profound exhaustion phenotype. Additionally, the flow cytometry analysis of exhaustion-related markers further supports this finding (Figure S1B). Furthermore, it is worth noting that the downregulation of early exhaustion-related genes and upregulation of late exhaustion genes observed in round 3 M28Z CAR-T cells compared to earlier rounds (Figure 1E) imply a progression toward a more severe exhaustion phenotype. GSEA analysis suggests a potential trend where round 2 M28Z cells display relatively elevated expression of genes linked to progenitor exhaustion and relatively reduced expression of genes associated with terminal exhaustion compared to round 3 M28Z cells (Figure 1F). While these differences are not statistically significant, the data indicate that M28Z CAR-T cells might progressively shift from a progenitor to a terminally exhausted phenotype as they encounter increasing rounds of tumor cell challenges.

In addition, Gene Ontology (GO) enrichment analysis revealed a downregulation of genes involved in metabolic pathways in both progenitor and terminally exhausted T cells compared to the activated state (Figure S1C), consistent with previous studies.^43^^,^^44^ Taken together, these results indicate that hypofunctional CAR-T cells that undergo multiple rounds of tumor challenge exhibit profound T cell exhaustion phenotypes.

### Anti-PD-1/PD-L1 blockade functionally reinvigorates hypofunctional CAR-T cells

To evaluate the effect of anti-PD-1/PD-L1 blockade on CAR-T cells and determine whether it could restore their tumor-killing ability in this model, we first confirmed the expression of PD-L1 and PD-1 in tumor cells and hypofunctional CAR-T cells, respectively (Figures S1D and S1E). For CAR-T cells treated with different rounds of tumor challenge, we then assessed the effect of anti-PD-1/PD-L1 blockade (Figure S1F). The tumor-killing capability of CAR-T cells after 2 rounds of tumor challenge was significantly improved (Figure 1G). M28Z from round 1 achieved complete lysis of tumors and therefore was not in hypofunctional state, and round 3 CAR-T cells seemed to be too exhausted to be rescued by anti-PD-1/PD-L1 blockade (Figures 1A and 1G). These results are consistent with recent studies demonstrating that progenitor-exhausted T cells can respond to PD-1 blockade, while terminally exhausted cells cannot.^11^^,^^12^^,^^13^^,^^14^

After validation with multiple donors, we found that CAR-T cells exhibited a progenitor exhausted-like state when the tumor-killing percentage ranged between 40% and 60%, usually after 2–3 rounds of tumor stimulation (Figure S1G). In this state, CAR-T cell efficacy was significantly restored by the anti-PD-1/PD-L1 antibody in all donors (Figures 1H and S1H). However, when the tumor-killing percentage dropped below 40% after 3–4 rounds of tumor stimulation, the CAR-T cells were in a terminally exhausted-like hypofunction state that could not be rescued by PD-1 antibody treatment (Figures S1I and S1J). These findings highlight the potential of this model as an *in vitro* screening platform to identify compounds or antibodies that restore the function of hypofunctional CAR-T cells.

### Screening an FDA-approved compound library identified miltefosine as a functional enhancer of hypofunctional CAR-T cells

**Figure 2:**
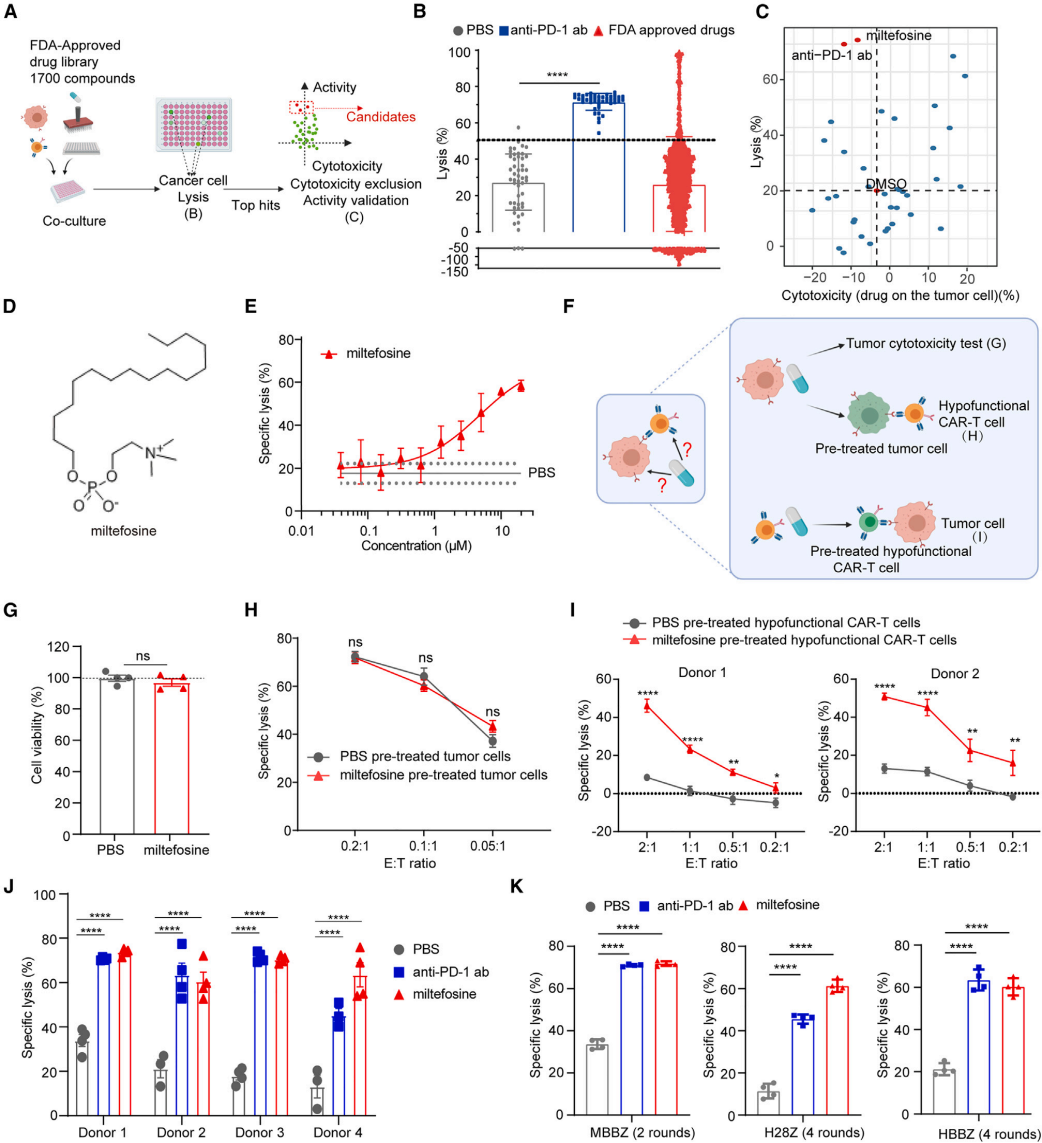
FDA-approved compound library screening identified miltefosine as a potent enhancer of hypofunctional CAR-T cell function (A) Workflow of high-throughput drug screening using hypofunctional CAR-T cell model. (B) The results of primary screening. (C) The results of secondary screening. (D) The chemical structure of miltefosine. (E) Dose-response analysis of miltefosine’s effect on the specific lysis of NCI-H226-luciferase after coculture with hypofunctional CAR-T cells (*n* = 4). (F) The schematic diagram of experimental design of (G–I). (G) Cell viability of NCI-H226-luciferase cells after miltefosine treatment (*n* = 4). (H) M28Z-mediated specific lysis of NCI-H226-luciferase cells with miltefosine or PBS pretreatment for 4 days (*n* = 4). (I) Hypofunctional M28Z were pretreated with miltefosine or PBS for 4 days and then cocultured with NCI-H226-luciferase cells at different E:T ratios (*n* = 4). (J) The specific lysis of NCI-H226-luciferase cells after coculture with hypofunctional M28Z with miltefosine or anti-PD-1 antibody treatment for 4 days at 1:1 E:T ratio (*n* = 4, results of other E:T ratios shown in Figure S2A). (K) The specific lysis of NCI-H226-luciferase cells cocultured with hypofunctional MBBZ (after stimulated by NCI-H226-luciferase cells 2 rounds at 1:1 ratio), H28Z (after stimulated by NCI-H226-luciferase cells 4 rounds at 1:1 ratio), and HBBZ (after stimulated by NCI-H226-luciferase cells 4 rounds at 1:1 ratio) CAR-T cells for 4 days with miltefosine or anti-PD-1 antibody treatment (*n* = 4). Unpaired t test was used in statistical analysis. NS, not significant, ∗*p* < 0.05, ∗∗*p* < 0.01, ∗∗∗∗*p* < 0.0001. All error bars denote SEM. See also Figure S2.

To identify effective compounds that rejuvenate hypofunctional CAR-T cells, we screened an FDA-approved drug library containing 1,700 compounds (at a concentration of 10 μM) using a coculture system of progenitor exhausted-like hypofunctional CAR-T cells and tumor cells. DMSO and anti-PD-1 antibody were used as the solvent and positive control, respectively. The specific lysis of cancer cells was measured as an indicator of CAR-T cell-mediated cytotoxicity (Figure 2A). In the primary screen, 181 compounds were found to significantly enhance cancer cell lysis and were selected for further confirmation (Figure 2B).

To exclude compounds that directly kill tumor cells, we assessed their cytotoxicity in tumor cells separately. As a result, we selected 38 compounds that showed no significant cytotoxicity against tumor cells for further validation. In the secondary screen, we found that miltefosine significantly enhanced the specific cancer lysis capability of hypofunctional CAR-T cells without overt cytotoxicity (Figures 2C and 2D).

We selected a working concentration of 15 μM based on its dose-response curve (Figure 2E). As miltefosine has been used as an antineoplastic agent,^45^ we evaluated its effect on tumor cells and hypofunctional CAR-T cells independently (Figure 2F). The results confirmed that miltefosine had no direct cytotoxicity in tumor cells at the working concentration (Figure 2G). To test the possibility that miltefosine sensitizes tumor cells to CAR-T cell-mediated lysis, we pretreated tumor cells with or without miltefosine for 4 days and then cocultured them with hypofunctional CAR-T cells at different E:T ratios. The results showed that miltefosine had no significant effect on tumor cell sensitivity to CAR-T cells (Figure 2H). Furthermore, to confirm that miltefosine directly affects CAR-T cells, hypofunctional CAR-T cells were first treated with miltefosine for 4 days and these pretreated CAR-T cells were then cocultured with tumor cells in the absence of miltefosine. Impressively, the hypofunctional CAR-T cells pretreated with miltefosine showed significant improvement in efficacy (Figure 2I).

Miltefosine’s effect was further validated using T cells from different donors (Figures 2J, S2A, and S2B). This effect was not specific to M28Z CAR-T cells, as similar results were observed with mesothelin-4-1BB (MBBZ) and HER1 CAR-T cells using either CD28 (H28Z) or 4-1BB (HBBZ) costimulatory domains (Figure 2K). Furthermore, miltefosine enhanced the efficacy and interferon (IFN)-γ secretion of CAR-T cells in the low E:T ratio hypofunction assay as well (Figures S2C and S2D).

In addition to acting on hypofunctional CAR-T cells, miltefosine may also modulate the function of unmodified T cells under exhaustion-inducing conditions. To test this hypothesis, we evaluated the effect of miltefosine on exhausted mouse antigen-specific T cells and human T cells. Similar to the results observed in human CAR-T cells, the tumor-killing effect of mouse OT-1 T cells was significantly reduced after multiple rounds of OVA^+^ B16F10 cell challenge, whereas miltefosine treatment rescued this effector defect in OT-1 T cells from two independent donors (Figure S2E). Exhausted human T cells were prepared following the method of a previous study,^32^ by chronic stimulation with CD3 antibody (Figure S2F). Miltefosine-treated exhausted T cells showed higher expression levels of effector-related genes after phorbol 12-myristate 13-acetate (PMA) and ionomycin stimulation (Figure S2G).

In addition, we treated fresh CAR-T cells with miltefosine and found no effect (Figure S2H), indicating that miltefosine only acts on exhausted CAR-T cells. As CAR-T cell exhaustion progressed, we found that the proportion of CD8^+^ cells increased (Figure S2I), consistent with previous reports.^46^ Interestingly, miltefosine treatment increased the proportion of CD4^+^ cells in exhausted CAR-T cells (Figure S2J). Furthermore, we isolated hypofunctional CD4^+^ cells and found that miltefosine could restore their function as well (Figure S2K).

Taken together, our results indicate that miltefosine directly enhances the function of hypofunctional CAR-T cells, and this effect has been validated across multiple donors, diverse CAR-T cell types, and different T cell hypofunction models.

### Miltefosine enhances CAR-T cell efficacy independent of PD-1/PD-L1 pathway

**Figure 3:**
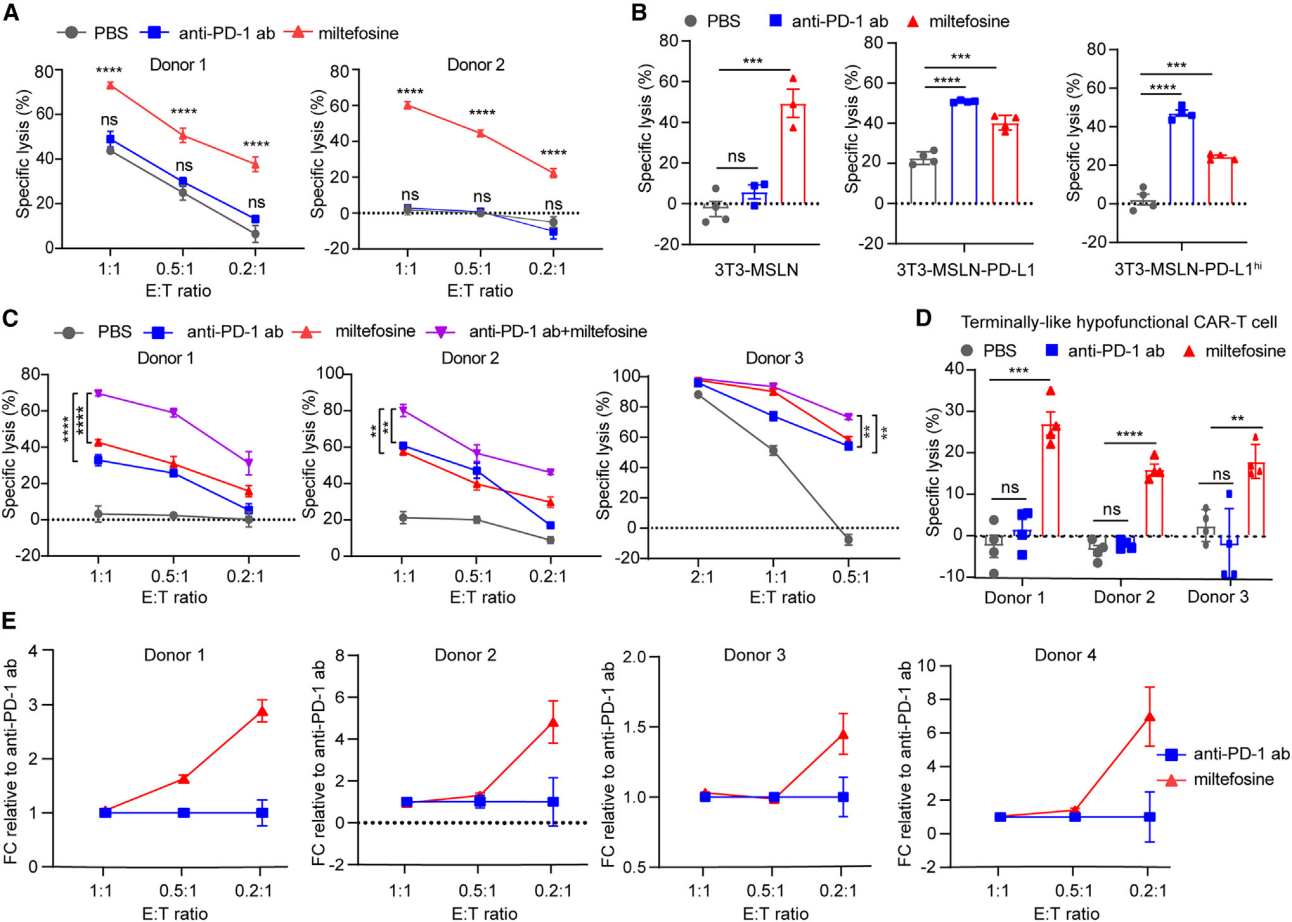
Miltefosine enhances CAR-T cell function in a PD-1/PD-L1 pathway-independent manner (A) The specific lysis of NCI-H226-luciferase cocultured with hypofunctional M28Z-PKO for 4 days with miltefosine or anti-PD-1 antibody treatment. M28Z-PKO, M28Z CAR-T cells with *PDCD1* knockout (*n* = 4). (B)The specific lysis of 3T3-MSLN-luciferase, 3T3-MSLN-PD-L1-luciferase, and 3T3-MSLN-PD-L1hi-luciferase cocultured with hypofunctional M28Z for 4 days with miltefosine or anti-PD-1 antibody treatment (*n* = 4). (C) The specific lysis of NCI-H226 cocultured with hypofunctional M28Z for 4 days with miltefosine or anti-PD-1 antibody treatment in three donors (*n* = 4). (D) The specific lysis of NCI-H226-luciferase cells cocultured with terminally exhausted-like M28Z CAR-T cells for 4 days with miltefosine or anti-PD-1 antibody treatment at a 1:1 ratio in three donors (*n* = 4). (E) The fold change (FC) of tumor killing with miltefosine treatment relative to anti-PD-1 antibody treatment (*n* = 4, related to Figure S2A). Unpaired t test was used in statistical analysis. NS, not significant, ∗∗*p* < 0.01, ∗∗∗*p* < 0.001, ∗∗∗∗*p* < 0.0001. All error bars denote SEM. See also Figure S3.

Given that PD-1/PD-L1 blockade is the most well-studied mechanism and successful strategy for enhancing the efficacy of exhausted T cells, we tested whether the effect of miltefosine on hypofunctional CAR-T cells is dependent on the PD-1/PD-L1 pathway. To test this, we generated CAR-T cells with *PDCD1* gene (encoding PD-1) knockout (M28Z-PKO) (Figure S3A) and found that miltefosine could still enhance the effector function (Figure 3A). As expected, since the CAR-T cells lack PD-1 expression, the anti-PD-1 antibody could not enhance their effector function (Figure 3A). On the other hand, we overexpressed mesothelin in NIH/3T3 cell lines, which do not express PD-L1. We then created several cell lines with different levels of PD-L1 expression (none, moderate, or high) (Figure S3B). We found that miltefosine improved the function of hypofunctional CAR-T cells regardless of the PD-L1 expression levels of the tumor cells (Figure 3B). In contrast, anti-PD-1 blockade only enhanced CAR-T cell function when PD-L1-expressing tumor cells were used (Figure 3B). These results indicate that miltefosine improves the efficacy of hypofunctional CAR-T cells in a PD-1/PD-L1-independent manner (Figure S3C) and suggest that miltefosine may have therapeutic benefits in the tumor microenvironment in which the PD-1/PD-L1 blockade is nonfunctional.

Next, we tested whether the combination of miltefosine and an anti-PD-1 antibody would yield a more significant functional outcome, in a scenario where CAR-T cells were in a progenitor-like exhausted state and PD-1 antibody treatment was effective. Our results showed that the combination therapy outperformed single-drug treatments in cells from various donors (Figure 3C). Notably, in the terminally exhausted state where PD-1 antibody treatment was ineffective, such as CAR-T cells from donor 1 after 3 rounds of tumor stimulation (Figures 1F and 1G), miltefosine was still able to enhance CAR-T cell activity (Figure 3D). Consistent with this, we found that as the E:T ratio decreased and the exhaustion level of CAR-T cells increased, the effect of miltefosine was even greater than that of anti-PD-1 antibody (Figure 3E), highlighting its potential as an effective therapy for T cells at various exhausted levels.

### scRNA-seq revealed that miltefosine treatment shifted CAR-T cell population

**Figure 4:**
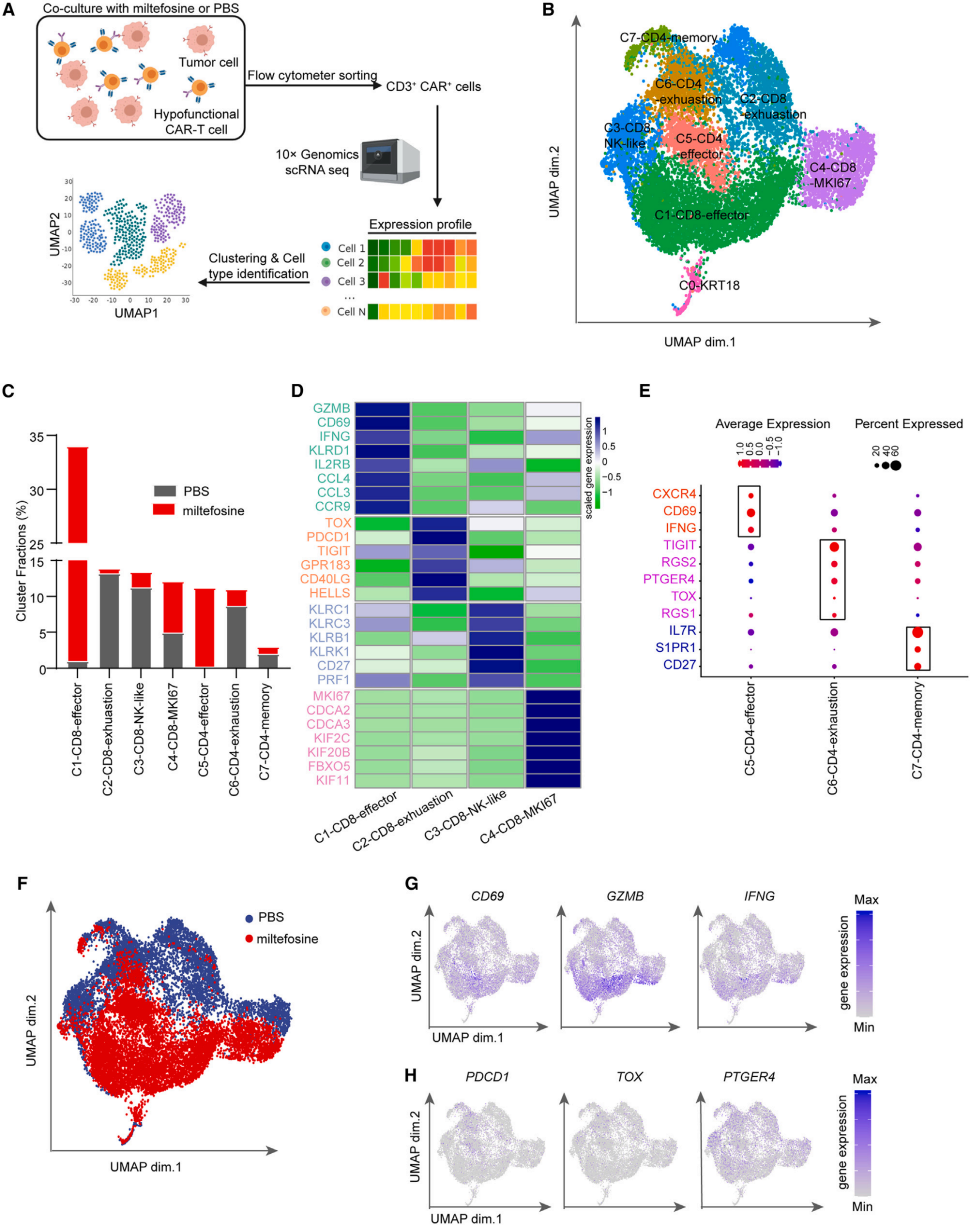
scRNA-seq identifies a distinct subpopulation shift of hypofunctional CAR-T cells upon miltefosine treatment (A) Schematic diagram of single-cell sequencing sample preparation and analysis. (B) UMAP projection of scRNA-seq data for each cluster. (C) Percentage of each cluster in total cells. (D) Heatmap of marker genes for CD8^+^ clusters defined in UMAP projection. (E) Heatmap of marker genes for CD4^+^ clusters defined in UMAP projection. (F) UMAP projection of scRNA-seq data from miltefosine and PBS samples. (G) Expression of activation-related genes shown in UMAP space. (H) Expression of exhaustion-related genes shown in UMAP space. See also Figure S4.

To characterize the landscape of hypofunctional CAR-T cells before and after miltefosine treatment in the coculture system described in Figure 2J, we performed scRNA-seq analysis (Figure 4A). Subsequently, we utilized a nonlinear dimensionality reduction technique (uniform manifold approximation and projection, UMAP) to analyze the data. Through unsupervised clustering analysis, we identified four unique clusters of CD8^+^ CAR-T cells, three stable clusters of CD4^+^ CAR-T cells (Figures 4B and S4A), and one cluster for tumor cells (cluster 0-KRT18, representing 1.2% of total cells [Figures 4B and S4B]). Each cluster displayed its distinct signature gene expression.

For CD8^+^ CAR-T cells (Figures 4C, 4D, and S4C), cluster 1 (C1-CD8-effector) mainly consisted of CAR-T cells from the miltefosine group and specifically expressed effector marker genes such as *GZMB*, *IFNG*, and *CD69*. The second cluster (C2-CD8-exhaustion) was distinguished by transcripts encoding *PDCD1* and *TOX*, which are T cell exhaustion-specific genes, and primarily contained CAR-T cells from the PBS group. Consistent with cluster 2, the third cluster (C3-CD8-NK-like), characterized by the specific expression of the natural killer (NK) cell receptor, also prevailed in the PBS group. This cluster has been reported to display NK-like cell transition in CAR-T cell dysfunction.^47^ The fourth cluster (C4-CD8-MKI67), expressing genes encoding MKI67, was equally distributed between the miltefosine and PBS groups. This result suggests that miltefosine treatment notably reduces the number of exhausted T cells and increases the population of functional CD8^+^ effectors.

Regarding CD4^+^ CAR-T cells (Figures 4C and 4E), the C5-CD4-effector cluster expressed genes encoding *CXCR4*, *CD69*, and *IFNG*, while the C6-CD4-exhaustion cluster was distinguished by exhaustion-specific genes (*TOX*, *TIGIT*, and *RGS2*). In concordance with CD8^+^ CAR-T cells, CAR-T cells from the miltefosine group were predominantly found in the C5-CD4-effector cluster, whereas CAR-T cells from the PBS group were enriched in the C6-CD4-exhaustion cluster. A smaller cluster (C7-CD4-memory, representing 2% of total cells) expressed genes encoding memory cell markers (*IL7RA* and CD27). Consistent with its effect on CD8^+^ cells, miltefosine treatment reduces the number of exhausted cells and increases the population of functional CD4^+^ T cells.

Consistent with unsupervised clustering analysis, the activation markers (*CD69*, *GZMB*, and *IFNG*) were principally expressed in the miltefosine group (Figures 4F, 4G, and S4D). Conversely, exhaustion-specific genes (*PDCD1*, *TOX*, and *PTGER4*) were slightly upregulated in the PBS group (Figures 4F, 4H, and S4D). These findings highlight that miltefosine-treated CAR-T cells exhibit improved effector capabilities and heightened resistance to exhaustion. Furthermore, Kyoto Encyclopedia of Genes and Genomes (KEGG) analysis indicated that metabolism pathways were upregulated in the miltefosine group (Figure S4E).

Our scRNA-seq analysis indicates that miltefosine improves effector function, diminishes exhaustion, and enhances metabolic activity in hypofunctional CAR-T cells. These results imply that miltefosine treatment shifts the CAR-T cell population toward a functional state that is further away from exhaustion.

### Miltefosine boosts the impaired glycolytic metabolism and glucose uptake of hypofunctional CAR-T cells

**Figure 5:**
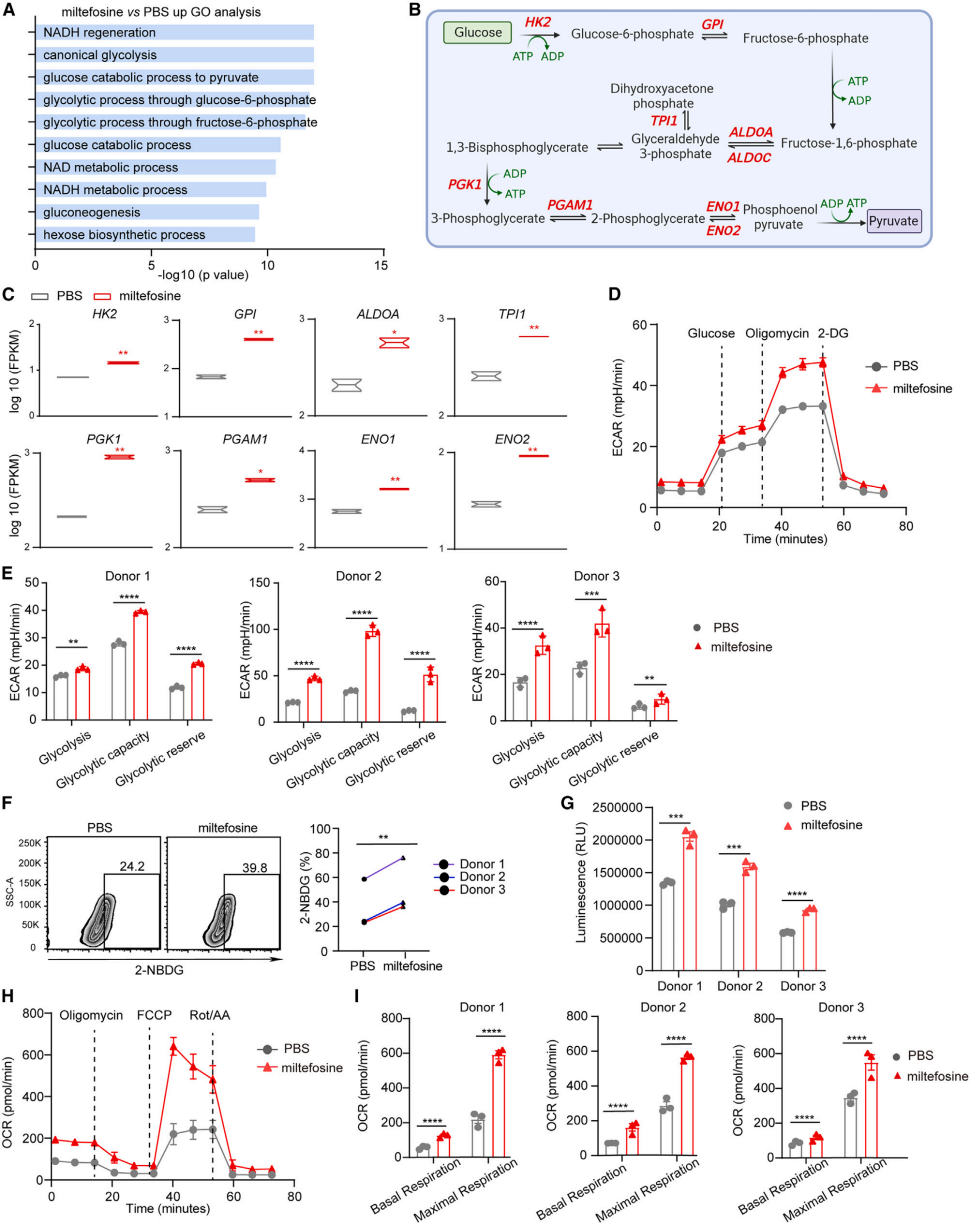
Miltefosine reverses the impaired glycolytic metabolism and glucose uptake of hypofunctional CAR-T cells (A) GO analysis of upregulated genes in miltefosine-treated hypofunctional CAR-T cells compared to PBS-treated samples. (B) The function of upregulated glycolysis-related genes in the glucose metabolism pathway. (C) Log_10_ FPKM values of glycolysis-related genes in hypofunctional CAR-T cells treated with miltefosine or PBS. (D) Changes in the extracellular acidification rate (ECAR) of donor 1-derived hypofunctional CAR-T cells after miltefosine treatment (*n* = 3). (E) Miltefosine improved the glycolytic ability of hypofunctional CAR-T cells derived from three different donors (*n* = 3). (F) 2-NBDG uptake in hypofunctional CAR-T cells treated with PBS or miltefosine. Each line represents a donor (*n* = 3). (G) Glucose uptake in hypofunctional CAR-T cells treated with PBS or miltefosine, measured using the Glucose Uptake-Glo assay (*n* = 3). (H) Changes in the oxygen consumption rate (OCR) of donor 1-derived hypofunctional CAR-T cells after miltefosine treatment (*n* = 3). (I) Miltefosine improved the OCR of hypofunctional CAR-T cells derived from three different donors (*n* = 3). Unpaired t test was used in statistical analysis. ∗*p* < 0.05, ∗∗*p* < 0.01, ∗∗∗*p* < 0.001, ∗∗∗∗*p* < 0.0001. All error bars denote SEM. See also Figure S5.

While scRNA-seq analysis provides valuable insights into the transcriptomic changes induced by miltefosine treatment at the single-cell level, the transcriptome sequencing depth is limited. Additionally, the coculture conditions may confound whether the observed changes are a direct effect of miltefosine or a consequence of functional recovery. To address this, we conducted bulk RNA-seq analysis on miltefosine-treated hypofunctional CAR-T cells (Figure S5A), in which the effect of miltefosine was previously confirmed (Figure 2I). GO enrichment analysis revealed that the differentially expressed genes (DEGs) upon miltefosine treatment were significantly enriched in glycolysis-related GO terms (Figure 5A). Notably, treatment with miltefosine led to the upregulation of a series of genes that encode metabolic enzymes related to glycolysis, including *HK2*, *GPI*, *ALDOA*, *TPI1*, *PGK1*, *PGAM1*, *ENO1*, *ENO2*, and others (Figures 5B and 5C). These genes are involved in almost every key step of the glycolysis pathway (Figure 5B), and their expression was significantly downregulated in CAR-T cells after multiple rounds of tumor challenge (Figure S5B). Furthermore, the results of real-time quantitative PCR showed that miltefosine-treated exhausted mouse OT-1 cells and human T cells also upregulated these glycolysis-related genes (Figures S5C and S5D). These findings demonstrate that miltefosine upregulates glycolysis-related genes in hypofunctional CAR-T cells and T cells.

To further investigate whether the upregulation of glycolysis-related genes leads to alterations in glycolytic metabolism in miltefosine-treated CAR-T cells, we performed a glycolytic stress test using the Seahorse XF analyzer. Glycolytic parameters were calculated by monitoring changes of the extracellular acidification rate (ECAR) in response to sequential addition of glucose to assess glycolysis, oligomycin to measure glycolytic capacity, and 2-deoxy-glucose (2-DG) to measure glycolytic reserve capacity. As shown in Figure 5D, miltefosine-treated hypofunctional CAR-T cells revealed a higher overall ECAR, reflecting a stronger glycolysis capacity. Hypofunctional CAR-T cells from three different healthy donors treated with miltefosine all showed a marked increase in basal glycolysis, oligomycin-stimulated glycolytic capacity, and glycolytic reserve capacity compared to PBS-treated cells (Figures 5D and 5E).

In aerobic glycolysis, glucose is the starting material, while glucose uptake is the major limiting factor. Miltefosine induced elevated glycolytic metabolism in hypofunctional CAR-T cells, which might be attributed to increased glucose availability.^43^^,^^48^ To validate this, the fluorescent glucose analog 2-NBDG was used to assess cellular glucose uptake in CAR-T cells. While hypofunctional CAR-T cells exhibited lower 2-NBDG staining compared with the “fresh” and “activated” cells (Figure S5E), miltefosine treatment enhanced 2-NBDG staining in three independent donor-derived hypofunctional CAR-T cells (Figure 5F). Given the question regarding the specificity of 2-NBDG as a measure of glucose transport,^49^ we further evaluated glucose transport using a bioluminescence detection method and confirmed that miltefosine treatment indeed enhanced glucose uptake (Figure 5G).

Given the observed increase in glucose uptake, we investigated whether OXPHOS, another major pathway of glucose metabolism, might also be affected. GSEA using the OXPHOS gene set showed a trend toward elevated expression of these genes in the miltefosine-treated group compared to the PBS-treated group, implying that miltefosine may also influence the OXPHOS pathway in hypofunctional CAR-T cells (Figure S5F). Moreover, we quantified the oxygen consumption rate (OCR) of hypofunctional CAR-T cells and found that miltefosine-treated cells exhibited a stronger mitochondrial OXPHOS capacity (Figures 5H and 5I). The ratios of OCR to ECAR were approximately equal (Figure S5G). These results suggest that miltefosine reinvigorates the bioenergetic state of hypofunctional CAR-T cells, enhancing both glycolysis and OXPHOS capability.

### Miltefosine’s effect is dependent on GLUT1

**Figure 6:**
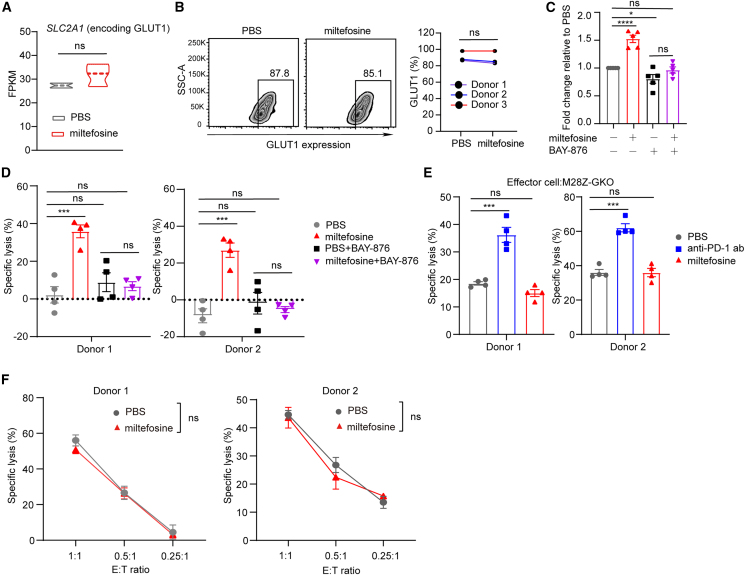
Miltefosine utilizes GLUT1 to augment glucose uptake in hypofunctional CAR-T cells (A) FPKM of *SLC2A1* in the PBS and miltefosine groups. (B) GLUT1 expression in hypofunctional CAR-T cells treated with PBS or miltefosine. Each dot represents a donor (*n* = 3). (C) 2-NBDG uptake in hypofunctional CAR-T cells treated with PBS or miltefosine combined with BAY-876, derived from five donors. Each dot represents a donor (*n* = 5). (D) Hypofunctional M28Z cells were pretreated with miltefosine or PBS combined with BAY-876 for 4 days and then cocultured with NCI-H226-luciferase cells at different E:T ratios (*n* = 4). (E) Specific lysis of NCI-H226-luciferase cells after coculture with hypofunctional M28Z-GKO cells treated with miltefosine or an anti-PD-1 antibody for 4 days at a 1:1 E:T ratio (*n* = 4). (F) Hypofunctional M28Z-GKO cells were pretreated with miltefosine or PBS for 4 days and then cocultured with NCI-H226-luciferase cells at different E:T ratios (*n* = 4). Unpaired t test was used in statistical analysis. NS, not significant, ∗*p* < 0.05, ∗∗∗*p* < 0.001, ∗∗∗∗*p* < 0.0001. All error bars denote SEM. See also Figure S6.

Cellular glucose uptake requires the members of the glucose transporter family (GLUTs), and GLUT1 plays the most significant role in glucose uptake of T cells.^50^^,^^51^^,^^52^ We observed that GLUT1 expression increased in CAR-T cells upon stimulation by tumor cells (Figures S6A–S6C), consistent with previous reports.^51^ However, at the RNA and protein levels, miltefosine did not alter GLUT1 expression in hypofunctional CAR-T cells (Figures 6A, 6B, and S6D). This led us to investigate whether miltefosine affects glucose uptake by modulating GLUT1 function. Indeed, miltefosine treatment significantly increased glucose uptake in hypofunctional CAR-T cells, but this effect can be abrogated by the GLUT1 inhibitor BAY-876 (Figures 6C and S6E). Similarly, the enhanced tumor-killing ability observed with miltefosine treatment was also abolished by BAY-876 (Figure 6D).

To further validate the connection between miltefosine and GLUT1, we conducted GLUT1 knockout (M28Z-GKO) in CAR-T cells by CRISPR-Cas9. The knockout efficiency was confirmed at the genetic (Figure S6F), mRNA (Figure S6G), and protein levels (Figure S6H). For the hypofunctional M28Z-GKO CAR-T cells, the anti-PD-1 antibody enhanced their cytotoxicity while miltefosine failed to do so (Figure 6E). Similarly, miltefosine pretreatment did not improve the efficacy of hypofunctional M28Z-GKO CAR-T cells (Figure 6F). Taken together, these results indicate that miltefosine promotes glucose availability in hypofunctional CAR-T cells in a GLUT1-dependent manner and thus improves their effector function.

### Miltefosine enhances the efficacy of CAR-T cells and T cells against solid tumor *in vivo*

**Figure 7:**
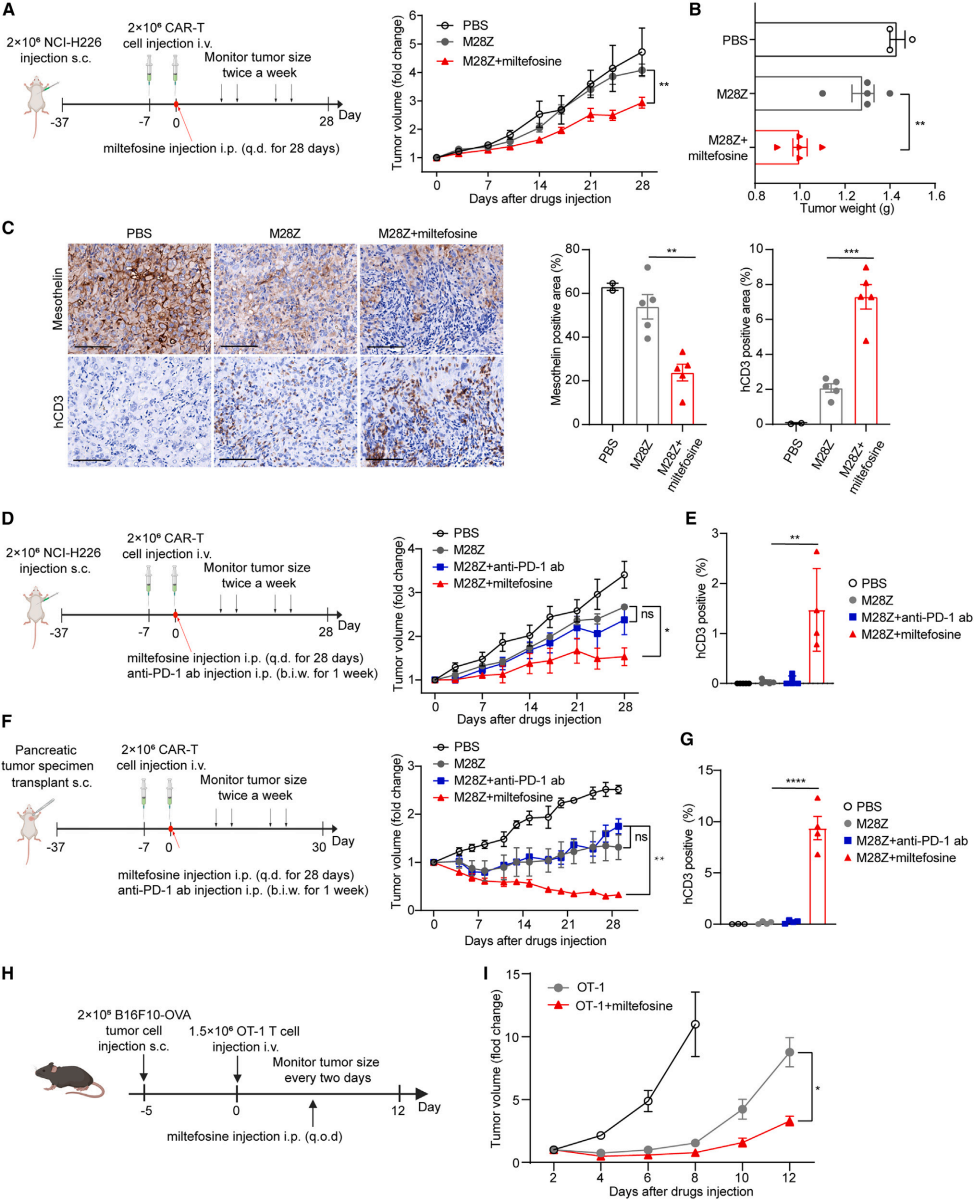
Miltefosine enhances CAR-T cell efficacy against solid tumors *in vivo* (A) Experimental timeline of CDX model and the fold change of tumor volume in CAR-T cell-treated CDX mouse model in the presence or absence of miltefosine administration (PBS group, *n* = 3; other groups, *n* = 5). (B) Tumor weight of each group at the end of experiment (PBS group, *n* = 3; other groups, *n* = 5). (C) The percentages of mesothelin- and hCD3-positive area in tumor tissues (PBS group, *n* = 2; other groups, *n* = 5). Scale bar: 200 μm. (D) The fold change of tumor volume in CDX mouse model treated with CAR-T cells derived from another donor, in the presence or absence of miltefosine or anti-PD-1 antibody treatment (*n* = 4). (E) The proportion of hCD3^+^ T cells in the peripheral blood of CDX mouse model (*n* = 4). (F) The fold change of tumor volume in CAR-T cell-treated PDX mouse model, in the presence or absence of miltefosine or anti-PD-1 antibody treatment (*n* = 4). (G) The proportion of hCD3^+^ T cells in the peripheral blood of PDX mouse model (*n* = 4). Unpaired t test was used in statistical analysis. (H) Experimental timeline of the OT-1-treated melanoma isograft tumor model. (I) The fold change in tumor volume in the OT-1-treated melanoma isograft tumor model in the presence or absence of miltefosine administration (*n* = 7). Unpaired t test was used in statistical analysis. NS, not significant, ∗*p* < 0.05, ∗∗*p* < 0.01, ∗∗∗*p* < 0.001, ∗∗∗∗*p* < 0.0001. All error bars denote SEM. See also Figure S7.

We further tested the efficacy-promoting effects of miltefosine on CAR-T cells *in vivo* using a cell line-derived xenograft (CDX) model. As shown in Figure 7A, M28Z CAR-T cells administered intravenously (i.v.) at this dosage did not exhibit significant tumor-suppressive effect *in vivo*; RNA-seq results suggested that tumor-infiltrating CAR-T cells in this model may exhibit signs of exhaustion (Figure S7A), which could imply that the activity of CAR-T cells is potentially being hindered by the solid tumor. In contrast, tumor volume (Figure 7A) measured over time and tumor weight (Figure 7B) upon sacrifice showed that miltefosine treatment enhanced antitumor efficacy of CAR-T cells. Then we detected the expression of mesothelin as a tumor cell marker and the expression of hCD3 as a CAR-T cell marker in tumor sections from these groups. We found a markedly decreased number of mesothelin-positive tumor cells and an increase in tumor-infiltrating CAR-T cells in the miltefosine-treated group compared with the M28Z group (Figure 7C).

Next, we tested the effect of miltefosine on CAR-T cells from another donor using this CDX model. Again, miltefosine-treated mice demonstrated a significant increase in antitumor efficiency of CAR-T cells, while anti-PD-1 antibody (pembrolizumab) treatment had no apparent effect (Figure 7D). This is consistent with what we observed in our *in vitro* results of terminal exhaustion-like hypofunctional CAR-T cells (Figure 3D) and suggests that miltefosine may function in the scenario in which an anti-PD-1 antibody has no effect in treating solid tumors. Higher CAR-T cell percentages in peripheral blood were found in miltefosine-treated mice than in PBS or anti-PD-1 antibody-treated mice (Figure 7E). As shown in Figure S7B, compared to control treatment, miltefosine treatment alone did not have a significant effect on tumor growth, indicating that miltefosine helped control tumor volume via CAR-T cells *in vivo*. To further explore the potential of combining miltefosine with anti-PD-1 antibody, we reduced the tumor burden of the CDX model and extended the observation time. The results showed that both miltefosine and anti-PD-1 antibody had noticeable effects when administrated individually, and the combined use of both treatments had a stronger impact on enhancing CAR-T function (Figure S7C).

Considering the heterogeneous nature of tumor in patients, we established a patient-derived xenograft model (PDX) of pancreatic carcinoma to further test the effect of miltefosine on CAR-T cells. Immunohistochemistry experiments showed heterogeneous mesothelin expression and negative PD-L1 expression in the pancreatic tumor specimens (Figure S7D). Miltefosine-treated mice almost completely cleared tumor and had higher CAR-T cell percentage in peripheral blood at the last time point, while anti-PD-1 therapy did not show any benefit compared with M28Z CAR-T cell (Figures 7F and 7G). Miltefosine alone did not affect the tumor growth (Figure S7E).

Furthermore, we evaluated the effect of miltefosine on tumor-specific OT-1 T cells in a syngeneic tumor model. A total of 2 × 10^5^ B16F10-OVA tumor cells were inoculated subcutaneously, 1.5 × 10^6^ OT-1 cells were transfused 5 days later, and then administration of miltefosine was initiated to observe changes in tumor volume (Figure 7H). As shown in Figure 7I, B16F10-OVA melanoma grew rapidly in untreated mice, and tumor volume continued to rise, quickly reaching the ethical limit score. Tumor-specific OT-1 T cells inhibited tumor growth, while miltefosine treatment further enhanced their efficacy. At the end of the experiment, the tumors in mice treated with miltefosine were significantly smaller than those in mice treated with OT-1 cells alone (Figure S7D). All these results demonstrate that miltefosine treatment endowed CAR-T and T cells with superior functions against solid tumor *in vivo*, demonstrating the promising potential in combining T cell-based immunotherapy with miltefosine in future clinical applications.

## Discussion

Despite promising results in the treatment of hematological malignancies, CAR-T cell therapy has limited clinical efficacy in treating solid tumors, and recent studies suggest that T cell exhaustion is a major contributor to treatment resistance.^53^ Although single-cell transcriptome analysis has well-characterized exhausted T cells in solid tumors,^15^^,^^16^^,^^17^ the underlying mechanisms remain unclear and require further investigation. A reliable experimental model that employs human primary T cells is necessary to fully dissect this process and develop effective interventions. Previously, we established a CAR-T cell hypofunction model by reducing the E:T ratio and prolonging the coculture time *in vitro*, which enabled the identification of key regulators of T cell exhaustion.^27^ However, the number of hypofunctional cells generated using this method is often limited. Repetitive stimulation of T cells by tumor cells or antigens is another classical method for evaluating T cell function and has been used in the development of T cell-based tumor immunotherapy.^11^^,^^54^^,^^55^^,^^56^^,^^57^^,^^58^ Recently, multiple groups have developed T cell dysfunction models using this strategy. Good and colleagues exposed CAR-T cells to serial target cell stimulation to mimic the T cell exhaustion process.^47^ Similarly, Belk et al. induced chronic T cell receptor (TCR) signaling *in vitro* by stimulating T cells with anti-CD3 and anti-CD28 antibodies,^32^ and Trefny and colleagues repetitively stimulated NY-ESO-1 TCR-T cells using human leukocyte antigen-A2-positive T2 tumor cells loaded with NY-ESO-1 peptides.^29^ In this study we established a method to generate many hypofunctional CAR-T cells by exposing M28Z CAR-T cells to multiple rounds of NCI-H226 tumor cell challenge and characterized their functional and transcriptomic features after each round of stimulation. By benchmarking the functional status, we were able to reproducibly generate progenitor exhausted-like and terminally exhausted-like CAR-T cells from different donors (Figures S1E–S1H). This allowed for high-throughput screening and validation of interventions that rescue the dysfunction of CAR-T cells at different exhaustion levels.

Blocking of immune checkpoints can reverse the inhibitory signal of T cells, and the combination of such checkpoint inhibitors with adoptive T cell therapy has demonstrated improved clinical efficacy.^59^^,^^60^ However, currently approved immune checkpoint inhibitors are typically monoclonal antibodies.^61^ Compared with antibody therapy, small molecule drugs offer numerous advantages for clinical application, including better penetration of solid tumor, lower production cost, oral bioavailability, and more convenience, which makes treatment opportunities available to more patients.^62^ Therefore, potent small molecule immunotherapeutics are highly desirable. Several studies have reported on small molecule drug screening for improving T cell function, using either functional competent CD19 CAR-T cells *in vitro*^63^ or the LCMV-CL13 chronic viral infection model.^64^ To directly address the impaired function of human exhausted T cells, we established a robust screening model based on the functionally impaired human primary T cells with defined hypofunction phenotype. We screened the FDA-approved small molecule library using progenitor exhausted-like CAR-T cells that respond to PD-1 blockade and identified miltefosine as a promising drug candidate. Furthermore, we generated CAR-T cells with a terminally exhausted phenotype, which could also be reinvigorated by miltefosine. While it is a simple coculture system, both the CAR-T cells and tumor cells can be further engineered by overexpressing or knocking out specific genes to model interacting pathways between T cells and tumor. Cytokines such as transforming growth factor β and IL-10 could also be added to this system to further model the inhibitory tumor microenvironment, providing a useful and flexible platform for screening immunotherapeutics.

Miltefosine, an orally available alkyl phospholipid, has gained approval from the FDA as an antiparasitic for treating leishmaniasis in patients aged over 12.^41^ Additional therapeutic benefits have been reported in trypanosomiasis and primary amebic meningoencephalitis.^65^ In addition, it was also implemented as an experimental antineoplastic agent.^45^ However, in the subsequent clinical studies, miltefosine failed to produce positive responses in patients with osteosarcoma, head and neck squamous cell carcinoma, and advanced colorectal cancer.^66^^,^^67^ Our study suggests that miltefosine may function as an immunomodulatory agent for T cell exhaustion, rather than a direct antitumor agent. The lack of clinical efficacy in treating tumor in early clinical studies could be due to the lack of consideration of the immune factors of enrolled patients. On the same note, nivolumab and pembrolizumab, both anti-PD-1 antibodies, demonstrated significantly different outcomes in clinical trials of non-small cell lung cancer (NSCLC) patients, which may be attributed to the differences in patient selection criteria based on PD-L1 expression levels (>5% or >50%).^68^ Therefore, for future clinical studies of miltefosine for treating solid tumors, patients with “hot tumors,” characterized by high levels of tumor T cell infiltration and specific molecular characteristics, should be prioritized.

To meet the energy demands needed, T cell activation is accompanied by a metabolic switch to aerobic glycolysis, thereby providing the energy required to maintain rapid cell growth, proliferation, and effector functions.^69^ In cancer and chronic infection-induced T cell exhaustion processes, bioenergetic deficiency, including glycolysis and OXPHOS, is considered one of the main features of exhaustion. In LCMV clone 13-infected mouse, the most commonly used model of T cell exhaustion, early exhausted T cells showed an overall suppression in glucose uptake and glycolysis capability.^43^ After coculture CD8^+^ T cells with leukemia cells *in vitro*, the capacity for glycolysis and OXPHOS were decreased with the downregulation of related genes.^70^ Another study showed that both human and mouse tumor-infiltrating CD8^+^ T cells have deficiencies in glycolysis metabolism due to ENO1 dysfunction.^48^ After multiple rounds of tumor cell challenge, we observed the same defect of glucose uptake and the expression of glycolysis-related genes (Figures S5B and S5E), indicating that the metabolic characteristics of our CAR-T cell hypofunction model resemble those of endogenous exhausted T cells. Impressively, miltefosine treatment restored the defects in glycolysis and OXPHOS metabolism in hypofunctional CAR-T cells (Figure 5).

GLUT1, also named glucose transporter member 1 (*SLC2A1*), has been demonstrated to be a predominant glucose transporter in glucose uptake of T cells.^48^^,^^50^^,^^51^^,^^52^ Recently, several papers have reported that overexpressing glucose transporters, including GLUT1, by genetic engineering can improve glucose metabolism and thus boost the efficacy of CAR-T therapy.^71^^,^^72^^,^^73^ In our study, we found that miltefosine treatment had no effect on GLUT1 expression (Figures 6A, 6B, and S6D). By pharmacologically and genetically inhibiting GLUT1, we demonstrated that miltefosine promotes glucose utilization and enhances the effector function of hypofunctional CAR-T cells related to GLUT1 function. It was previously reported that phosphorylation of serine 226 on GLUT1 promotes the translocation of GLUT1 to the plasma membrane, thereby enhancing the glucose transport capacity.^74^ However, we did not observe significant changes in the phosphorylation of GLUT1 at S226 residue by miltefosine treatment (data not shown). Therefore, it is worth to further investigate the molecular mechanism of action of miltefosine on GLUT1 function in the future.

In summary, we established a CAR-T cell hypofunction model for high-throughput drug screening, and discovered that miltefosine reinvigorated the impaired antitumor function of exhausted T cells via glycolysis and OXPHOS capability improvement. Our findings reveal the promising clinical potential of miltefosine as an immunotherapeutic drug for the treatment of tumors.

### Limitations of the study

One limitation of our study is the small number of sample replicates for GSEA and DEGs analyses (10,000 cells in scRNA-seq and 2–3 replicates in bulk RNA-seq), which may restrict our ability to detect statistically significant differences.

Notably, the GSEA enrichment analysis for progenitor and terminal exhaustion states in Figure 1F was not statistically significant. However, Figure 1E shows that round 3 cells tend more toward a late exhaustion state compared to round 2 cells, indicating that both exhibit signs of exhaustion, albeit to different degrees. Specifically, round 2 cells are more aligned with a progenitor exhaustion state, while round 3 cells are more terminally exhausted. The GSEA analysis suggests a trend where round 2 M28Z cells have elevated expression of genes linked to progenitor exhaustion and reduced expression of those associated with terminal exhaustion compared to round 3 M28Z cells (Figure 1F). Although these differences are not statistically significant, they imply that M28Z CAR-T cells may progressively shift from a progenitor to a terminally exhausted phenotype with increasing rounds of tumor cell challenges.

## Resource availability

### Lead contact

Further information and requests for resources and reagents should be directed to and will be fulfilled by the lead contact, Haoyi Wang (wanghaoyi@ioz.ac.cn).

### Materials availability

This study did not generate new unique reagents.

### Data and code availability

The accession number for the sequencing data generated in this paper is China National Center for Bioinformation/Beijing Institute of Genomics, Chinese Academy of Science (GSA-Human: HRA005964) that are publicly accessible at https://ngdc.cncb.ac.cn/gsa-human. This paper does not report original code. Any additional information required to reanalyze the data in this paper is available from the lead contact upon request.

## Acknowledgments

We thank Beijing Cord Blood Bank for providing cord blood samples, and we are grateful to Prof. Ting Chen for providing OT-1 mouse. All schematic diagrams in this paper were created with BioRender.com.

This work was supported by Beijing Municipal Science & Technology Commission, Administrative Commission of Zhongguancun Science Park (Z221100007922017 to H.W.); 10.13039/501100002858China Postdoctoral Science Foundation (2023TQ0351 and 2024M753221 to Xingying Zhang); the Postdoctoral Fellowship Program (Grade C) of 10.13039/501100002858China Postdoctoral Science Foundation (GZC20232645 to Xingying Zhang); Program of “Bingzhi Postdoctoral” in the 10.13039/501100011186Institute of Zoology, Chinese Academy of Sciences (to Xingying Zhang); Program of Special Research Assistant in the 10.13039/501100002367Chinese Academy of Sciences (to Xingying Zhang); 10.13039/501100012166National Key Research and Development Program of China (2019YFA0110000 to H.W.); the 10.13039/501100002367Chinese Academy of Sciences (ZDBS-LY-SM005 to H.W.); the 10.13039/501100001809National Natural Science Foundation of China (81773269 to H.W.); and the 10.13039/501100012226Fundamental Research Funds for the Central Universities (2024-JYB-XJSJJ010 to C.Z.).

## Author contributions

Xingying Zhang, C.Z., and H.W. designed the study. Xingying Zhang, C.Z., S.L., N.T., X.P., and Xiang Zhang performed the experiments. J.D. and G.F. performed bioinformatic analysis of RNA-seq and scRNA-seq data. H.W. conceived and supervised this project. Xingying Zhang, C.Z., and H.W. wrote the manuscript. D.L., Yao Wang, W.H., Yu Wang, and N.S.-C. edited the manuscript.

## Declaration of interests

The authors declare no competing interests.

## STAR★Methods

### Key resources table

**Table:** 

REAGENT or RESOURCE	SOURCE	IDENTIFIER
**Antibodies**		

Pacific Blue™ anti-human CD3 Antibody (clone HIT3a)	Biolegend	Cat#300329; RRID: AB_10552893
APC anti-human CD279(PD-1) Antibody (clone EH12.2H7)	Biolegend	Cat#329908; RRID: AB_940475
APC anti-human CD274 (PD-L1) Antibody (clone MIH1)	BD	Cat#563741; RRID: AB_2738399
Atezolizumab (anti-PD-L1)	Selleck	Cat# A2004
Pembrolizumab (anti-PD-1)	Selleck	Cat# A2005
CD3e Monoclonal Antibody (clone145-2C11), Functional Grade	eBioscience	Cat#16-0031-81; RRID: AB_468846
Rabbit anti-human mesothelin Antibody (clone EP140)	ZSGB-BIO	Cat#ZA-0579
Rabbit anti- human CD3 Antibody (clone EP41)	ZSGB-BIO	Cat#ZA-0503
GLUT1 flow cytometry antibodyAntibody (clone EPR3915)	Abcam	Cat#ab195359; RRID: AB_2714026
isotype control	Abcam	Cat#ab199091; RRID: AB_2637027
GAPDH Antibody	Affinity Biosciences	Cat# AF7021; RRID: AB_2839421
GLUT1 Antibody	Affinity Biosciences	Cat#AF5462; RRID: AB_2837946
PE anti-human CD223 (LAG-3) Antibody	Biolegend	Cat#369306; RRID:AB_2629592
APC-*anti*-human CD366(Tim-3)	Biolegend	Cat#345012; RRID:AB_2561718
PE anti-human CD279 (PD-1)Antibody	Biolegend	Cat#379209; RRID:AB_2922607
APC anti-human CD152 (CTLA-4) Antibody	Biolegend	Cat #349908; RRID: AB_10679122
APC Mouse anti-Human CD244 (2B4)	BD	Cat #562350; RRID: AB_11153502
PE Mouse anti-human CD160	BD	Cat#562118; RRID: AB_10894019
APC Mouse anti-Human CD272(BTLA)	BD	Cat#564800; RRID:AB_2738959
TIGIT Monoclonal Antibody (MBSA43), PE	eBioscience	Cat#12-9500-42; RRID: AB_10714831

**Chemicals, peptides, and recombinant proteins**		

Fetal Bovine Serum, qualified, One Shot™ format, Australia Gibco™	Gibco	Cat#A3161001
Human mononuclear cell separation fluid	DongFang HuaHui Biomedical Technology	Cat#25710
ACK Lysis Buffer	Thermo Fisher	Cat#A1049201
Dynabeads™ Human T-Activator CD3/CD28 for T cell Expansion and Activation	Thermo Fisher	Cat#11131D
Recombinant human IL-2 protein	Sino Biological Inc	Cat#GMP-11848-HNAE
L-Glutamine	Gibco	Cat#25030081
Penicillin-Streptomycin	Thermo Fisher	Cat#15140-122
Trypsin-EDTA (0.25%)	Gibco	Cat#25200072
Lipofectamine™ 3000 Transfection Reagent	Thermo Fisher	Cat#L3000015
Steady-Glo® Luciferase Assay System	Promega	Cat#E2520
Corning® Matrigel® Matrix	Corning	Cat#354277
Fetal Bovine Serum	Gibco	Cat#10270106
CellTiter-Glo® 2.0 Cell Viability Assay	Promega	Cat#G9242
OVA_257-264_ peptide	Qiangyao Biotech Inc	Cat#1955-5-5
FDA-approved drug library	TargetMol	Cat#L1000
miltefosine	TargetMol	Cat#T0033
CD3e Monoclonal Antibody	eBioscience	Cat#16-0031-81
2-NBDG	Thermo Fisher	Cat#N13195
PMA+Ionomycin	DAKEWEI	Cat #2030421

**Critical commercial assays**		

EasySep^TM^ human T cell enrichment kit	STEMCELL Technologies	Cat#19051
Seahorse XFp Glycolysis Stress Test Kit	Agilent	Cat#103017-100
P3 Primary Cell 4D-Nucleofector X Kit	Lonza	Cat#V4XP-3024
RNeasy Mini Kit (50)	QIANGEN	Cat#74104
TransScript® One-Step gDNA Removal and cDNA Synthesis SuperMix	TransGen Biotech	Cat#AT311-03
Hieff qPCR SYBR Green Master Mix (No Rox)	Yeasen Biotech	Cat#11201ES08
Glucose Uptake-Glo™ Assay	Promega	Cat#J1341

**Deposited data**		

Bulk RNA and single cell RNA sequencing data	This paper	GSA-Human: HRA005964

**Experimental models: Cell lines**		

Human cell line: NCI-H226(lung cancer cell line)	ATCC	Cat#CRL-5826
Human cell line: 293T cell Line	ATCC	Cat#CRL-11268
Human cell line: NCI-H226-luciferase	Constructed by our lab	NA
Human cell line: NCI-H226-PDL1-luciferase	Constructed by our lab	NA
Mouse cell line: 3T3-mesothelin	Constructed by our lab	NA
Mouse cell line: 3T3-mesothelin-PDL1-luciferase	Constructed by our lab	NA
Mouse cell line: 3T3-mesothelin-PDL1^hi^-luciferase	Constructed by our lab	NA
Mouse cell line: B16F10-OVA	Constructed by our lab	NA

**Experimental models: Organisms/strains**		

Mouse: NPG (NOD.Cg-Prkdc^scid^ Il2rg^tm1Vst^/Vst)	Vitalstar	NA
Mouse: C57BL/6J	Vital River	NA
Mouse: OT-1 (C57BL/6-Tg(TcraTcrb)1100Mjb/J)	Gift from Prof. Ting Chen	NA
Tumor tissue: Patient-derived xenograft	Vitalstar	NA

**Recombinant DNA**		

Plasmid: pX330-SpCas9-HF1	Addgene	Cat#108301
Plasmid: pMD2.G	Addgene	Cat#12259
Plasmid: psPAX2	Addgene	Cat#12260
Plasmid: FUW-M2rtTA	Addgene	Cat#20342
Plasmid: FUW-EF1α-MBBZ-p2A-eGFP	Constructed by our lab	N/A
Plasmid: FUW-EF1α-M28Z-p2A-eGFP	Constructed by our lab	N/A
Plasmid: FUW-EF1α-H28Z-p2A-eGFP	Constructed by our lab	N/A
Plasmid: FUW-EF1α-HBBZ-p2A-eGFP	Constructed by our lab	N/A
Plasmid: pLKO-hPGK-OVA-IRES-tdTomato	A gift from Prof. Ting Chen	N/A
cDNA for full-length PDL1	Origene	N/A

**Software and algorithms**		

FlowJo 10	https://www.flowjo.com	N/A
GraphPad-Prism 8	https://www.graphpad.com	N/A
GSEA	http://www.gsea-msigdb.org/gsea/index.jsp	N/A
MACS2	https://pypi.org/project/MACS2/	N/A
Homer	http://homer.ucsd.edu/homer/	N/A
STAR	Bioconductor	N/A
Stringtie	Bioconductor	N/A
Cellranger	10X Genomics	https://www.10xgenomics.com/
Seurat	Satija Lab	satijalab.org/seurat/
Harmony	Bioconductor	bioconductor.org

**Other**		

DPBS	Gibco	Cat#C14190500CP
DMEM	Gibco	Cat#C11995500CP
RPMI 1640	Gibco	Cat#C11875500CP
X-VIVO15	Lonza	Cat#04-418Q
Opti-MEM™ I Reduced Serum Medium	Gibco	Cat#31985070

### Experimental model and study participant details

#### Cell lines and culture conditions

In this study, we used NCI-H226 cells that were positive for mesothelin, as well as HER1. For the luciferase-based cytolysis assay, we transduced tumor cell lines with lentivirus of firefly luciferase from Genechem (Shanghai, China) to produce NCI-H226-luciferase and 3T3-luciferase. The full-length cDNA of PD-L1 was obtained from Origene and subcloned into the FUW lentiviral backbone. The resulting PDL1 lentivirus was then transduced into NCI-H226-luciferase and 3T3-luciferase cells to produce NCI-H226-PD-L1-luciferase and 3T3-PDL1-luciferase. The NCI-H226-PD-L1-luciferase cells were sorted by flow cytometry (MoFlo XDP, Beckman Coulter Inc). We also obtained the full-length cDNA of mesothelin from Origene and subcloned it into the FUW lentiviral backbone. This mesothelin lentivirus was transduced into 3T3-luciferase and 3T3-PD-L1-luciferase cells to produce 3T3-MSLN-luciferase and 3T3-MSLN-PDL1-luciferase. The cells were also sorted by flow cytometry. We prepared the B16F10-OVA cell line, which is a murine B16F10 melanoma cell line expressing the chicken ovalbumin gene (OVA) containing the H2-Kb-restricted OVA_257-264_ epitope (SIINFEKL), through lentivirus transduction. All cell lines, including those obtained from ATCC, were cultured according to the manufacturer’s instructions. The NCI-H226 cells were cultured in RPMI 1640 supplemented with 10% heat-inactivated FBS, 100 U/mL penicillin, 100 mg/mL streptomycin sulfate, and 1% L-glutamine. The lentiviral producer cell line 293T was maintained in DMEM supplemented with 10% heat-inactivated FBS, 100 U/mL penicillin, 100 mg/mL streptomycin sulfate, and 1% L-glutamine. Finally, we confirmed that all cell lines used in this study were free from mycoplasma contamination by PCR.

#### Animals

Tumor xenograft studies were performed using NPG (NOD. Cg-Prkdcscid Il2rgtm1Vst/Vst) mice aged 6–8 weeks (Beijing Vitalstar Biotechnology Co.,Ltd.). Murine isograft tumor model was performed using 8-10-week-old C57BL/6J mice (Beijing Vital River Laboratory Animal Technology Co., Ltd.). OT-1 (C57BL/6-Tg (TcraTcrb)1100Mjb/J) mice aged 8–10 weeks were gifted by Ting Chen (National Institute of Biological Sciences, Beijing). All animals were raised in pathogen-free conditions at the animal facilities (Institute of Zoology, Chinese Academy of Sciences) and cared for in accordance with the International Association for Assessment and Accreditation of Laboratory Animal Care policies and certification.

#### Primary human T cells

With informed consent, we obtained peripheral blood mononuclear cells (PBMCs) from healthy volunteers at the Chinese PLA General Hospital (Beijing, China). We isolated primary human T cells using separation medium 1.007 (Beijing Dong Fang Hua Hui Biomedical technology co., Ltd, Cat#25710) and the EasySep human T cell enrichment kit (Stemcell Technologies, Cat#19051), following the manufacturer’s protocols. T cells were then stimulated using anti-CD3/CD28 Dynabeads (Thermo Fisher, Cat#11131D) at a 1:1 ratio. The isolated T cells were cultured in X-VIVO15 medium (Lonza) supplemented with 5% (v/v) heat-inactivated fetal bovine serum and 400 IU/mL recombinant human IL-2 (Sino Biological Inc., Cat#GMP-11848-HNAE). Cryopreservation was performed at a concentration of 2 × 10^7^ T cells per vial.

#### Study approval

All experiments involving animals were approved by the Animal Ethics Committee of the Institute of Zoology, Chinese Academy of Sciences (IOZ20190078).

### Method details

#### Generation of CAR constructs

In this study, we employed CAR constructs, as previously described.^27^ Briefly, the structure of M28Z was incorporated with P4 scFv, eGFP, as well as the CD28 and CD3-zeta signaling domains. MBBZ was incorporated with P4 scFv, eGFP, as well as the 4-1BB and CD3z signaling domains. H28Z CAR was incorporated with HER1 scFv, eGFP, as well as the CD28 and CD3-zeta signaling domains. HBBZ CAR was incorporated with HER1 scFv, eGFP, as well as the 4-1BB and CD3z signaling domains. CAR^+^ T cells were identified by eGFP expression.

#### Productions of viral vector and CAR-T cells

To produce the CAR lentiviral supernatant for this study, we utilized the 293T packaging cell line and followed previously established methods.^27^ Briefly, we co-transfected 12 μg of CAR plasmids, as well as 6μg each of pMD2.G and psPAX2 packing plasmids DNA into 293T cells using Lipofectamine 3000. This was performed when the 293T cells reached 95% confluency in 10-cm plates. After 48 h and 72 h post transfection, we harvested the virus supernatant and concentrated it through ultracentrifugation (Millipore) at 4000 rpm for 1 h. We then froze the concentrated virus at −80°C for future use. Isolated T cells were transfected by CAR lentiviral 24 h after activation using CD3/CD28 beads. 2 × 10^6^ T cells were resuspended in 1 mL T cell medium and transfected with 100 μL concentrated virus, 1 μL polybrene was added for increasing infection efficiency. The efficiency of CAR could be detected after 48 h.

#### Electroporation of CAR-T cells

CRISPR/Cas9 gene editing was performed by electroporation Cas9/gRNA (RNP) complex using 4D-Nucleofector System N (Lonza), Primary cell 4D-nucleofector kit (V4XC-1032, Lonza). RNP containing 6 μg Cas9 protein and 6 μg sgRNA were pre-complexed for 30 min at room temperature to create ribonucleoprotein complexes. 5×10^5^ CAR-T cells that were infected 48 h were centrifuged at 300 g for 5 min and resuspended in 20 μL transfection buffer, the RNP was also resuspended by 20 μL transfection buffer mentioned. Then transferred the mixture into the electroporation cuvette using the EO-115 protocol in 16-well cuvette strips. CAR-T cells were recovered in 200 μL preheated T cell medium and expanded as described above. Gene knockout efficiency was detected using TIDE (Tracking Indels by Decomposition) three days after electroporation. Subsequently, *in vitro* cell experiments were conducted seven days after electroporation. The sgRNA sequence of *PDCD1* is: gtctgggcggtgctacaact. The sgRNA sequence of *SLC2A1* is: ggatgctctccccatagcgg.

#### Luciferase-based cytolysis assay

We performed luciferase-based cytolysis assay as previously described.^75^ In brief, tumor cells were suspended at a density of 1×10^5^ cells/mL in RPMI 1640 medium, then 100 μL tumor cells were seeded in 96-well white opaque plate, and 100 μL effector cells were added in white opaque plate at required ratio and cultured for required time. At the end of experiment, 10 μL of Steady-Glo luciferase substrate (Promega, Cat#E2520) was added in white opaque plate for 5 min at room temperature. Luminescence was recorded by PerkinElmer VICTOR X3. The percentage of specific lysis was calculated as the equation (% killing = 100×(1 - (RLU from well with effector and target cell coculture))/(RLU from well with target cells)). When CAR-T cell function is impaired and there is no killing effect, the lysis may show a negative value due to slight variations in the number of tumor cells in each well.

#### Real time cell analysis (RTCA)-based cytolysis assay

B16F10-OVA tumor cells were suspended at a density of 1×10^5^ cells/mL in RPMI 1640 medium, then 100 μL tumor cells were seeded in 96-well plate (ACEA Bio, Cat#5232368001). 100 μL effector cells were added in plate at required ratio next day. The percentage of specific lysis was calculated as the equation (% killing = 100× (1 - (cell index from well with effector and target cell coculture)/cell index from well with target cells)).

#### Multiple rounds of tumor cells challenge

Tumor cells and effector cells were co-cultured at a 1:1 E:T ratio. After co-culture for 48 h, all tumor cells were lysed, CAR-T cells were collected and co-cultured with fresh tumor cells at 1:1 E:T ratio for 48 h. By the analogy, the experiment was finished until CAR-T cell cells losing cytolysis ability. The percentage of specific lysis was recorded based on luciferase assay every round co-culture.

#### Flow cytometry

Cells were harvested on the required times and stained according to antibody’s protocols. The stained samples were analyzed using FACS Arial II (BD Bioscience) and data were analyzed using FlowJo software.

#### Quantitative real-time PCR

Total RNA was extracted using RNAeasy Mini Kit (QIANEN, Cat#74104) and cDNA was generated using TransScript One-Step gDNA Removal and cDNA Synthesis SuperMix kit (TransGen, Cat#AT311-03). RT-qPCR was done using Hieff qPCR SYBR Green Master Mix (Yeasen Biotech, Cat#11201ES08). All primers used for qPCR are listed in Table S1.

#### Western blot

Whole cell lysates of CAR-T cells awere generated by lysing 5×10^6^ washed cells in 200 μL RIPA buffer containing standard protease inhibitors cocktail. Samples were incubated on ice for 10 min and then subjected to BCA analysis. 20 μg of total protein was used for western blot analysis. The following primary and secondary antibodies were used: anti-GLUT1 (Affinity Biosciences; Cat#AF5462; RRID: AB_2837946) and anti-GAPDH (Affinity Biosciences; Cat#AF5462; RRID: AB_2837946).

#### Bulk RNA-seq sample preparation

The CAR^+^CD8^+^ cells was sorted and sent to BIOMARK Technologies (Beijing, China). They accomplished RNA extraction, library building and sequencing. Except for the exhausted CAR-T cells treated with miltefosine or PBS (Figure 5), which had two replicates, all other groups had three replicates.

#### Bulk RNA-seq library preparation and sequencing

After quality control and quantification of the RNA, sequencing libraries were generated using KAPA Stranded mRNA-Seq Kit for Illumina Platforms according to the manufacturer’s recommendations. The indexed libraries were sequenced on an Illumina Hiseq 2500 platform and 100 bp/150 bp paired-end reads were generated.

#### Bulk RNA-seq analysis

The quality of raw paired-end sequencing reads was checked by FastQC (v0.11.9). Genomic alignment was performed using STAR (version = 2.7.7a) aligner to human reference genome (GRCm38/hg38). Stringtie (version 2.1.1) was employed to calculate the read counts of per gene. Differentially expressed genes between samples were identified by Deseq2 R package (version 1.30.1) with |Log2-fold change | >1 and *p*-value < 0.05 as thresholds. The DEGs were carried out Gene Ontology Pathway analysis and Kyoto Encyclopedia of Genes and Genomes analysis by clusterProfiler (3.14.3) and org.Hs.e.g.,.db (3.10.0) packages. Pheatmap (1.0.12) was used for the heatmap visualization. GSEA was performed with software GSEA (4.2.3). PCA was performed by R packages, including tidyr (1.1.2), dplyr (1.0.2) and ggplot2 (3.3.2).

#### ScRNA-seq sample preparation

Hypofunctional CAR-T cells (from one donor) were co-cultured with NCI-H226 with or without miltefosine (15 μM) treatment for 4 days, GFP^+^ and CD3^+^ CAR-T cells were sorted and sent to Annaroad Gene Technology (Beijing) Co., Ltd.

#### 10× single-cell library construction and sequencing

Single-cell suspensions were converted to barcoded scRNA-seq libraries according to standard protocols of the Chromium single-cell 3′ kit to capture 5000 to 10,000 cells/chip position (V2 chemistry). All the remaining procedures including the library construction were performed according to the standard manufacturer’s protocol. The libraries were applied to pair-end sequencing with read lengths of 150nt on Illumina HiSeq Xten platform.

#### ScRNA-seq data processing and determination of the major cell types

Droplet-based sequencing data were mapped to the GRCh37 human reference genome through Cell Ranger (version 4.0.0, 10x Genomics) to generate digital gene expression matrices. The data from all samples were read into the Seurat R package (version 4.0.0) for the further processing. Firstly, data filtering was conducted by retaining cells expressed 200 and 7500 genes inclusive, and had mitochondrial content less than 10 percent. Each library was scaled by library size and log-transformed (using a size factor of 10,000 molecules per cell). The top 3000 highly variable Genes (HVGs) were identified through the function “FindVariableFeatures.” Then all the datasets were integrated using the harmony R package. Merged data were scaled to unit variance and zero mean. The dimensionality of data was reduced by PCA. A K-nearest-neighbor graph was constructed based on the euclidean distance in PCA space using the “FindNeighbors” function and Louvain algorithm was applied to iteratively group cells together by “FindClusters” function with optimal resolution on the optimal principal components. Visualization was achieved by the UMAP. Finally, specific markers in each cluster were identified by the “FindAllMarkers” function and the Classic marker,the clusters were assigned to known cell types using the canonic markers. In addition, we filtered one clusters for their tiny cell number. Subclustering for major cell types was performed in the same way.

#### GLUT1 staining

For different states of CAR-T cells, take 100,000 cells and wash them twice with PBS. Then, add the antibody (Abcam; CAT#ab195359; RRID: AB_2714026) at a 1:200 dilution and incubate in the dark for 15 min. Wash the cells twice with PBS again before proceeding to flow cytometry analysis.

#### Glycolysis and mito stress test

Glycolysis and mitochondrial oxidative phosphorylation capability of CAR-T cells was analyzed using a Seahorse XFe96 analyzer (Agilent). Briefly, hypofunction CAR-T cells (sorted by flow cytometry) were cultured with or without Miltefosine (15 μM) for 96 h, then resuspended in XF assay medium and seeded in XF cell culture plate with 2×10^5^ cell per well. The plate was incubated in a CO^2^-free incubator at 37°C for 1 h and transferred to the XFe96 analyzer for glycolysis and mitochondria stress analysis. Glycolysis capability was measured via ECAR (mpH/min), with the use of real-time injections of glucose (10 mM), oligomycin (1 μM) and 2-deoxy-D-glucose (2-DG, 50 mM). Mitochondrial oxidative phosphorylation capability was measured via OCR (pmol/min), with the use of real-time injections of oligomycin (1.5 μM), FCCP (1.5 μM) and rotenone/antimycin A (Rot/AA, 0.5 μM). ECAR and OCR values were calculated using the Wave Controller program.

#### Glucose uptake test

Test by 2-NBDG, hypofunctional CAR-T cells (sorted by flow cytometry) were cultured for 4 days in the presence or absence of miltefosine (15 μM) or BAY-876 (1 μM). Afterward, the cells were washed with PBS and subjected to a 30-min incubation at 37°C with 5% CO_2_ in glucose-free DMEM supplemented with 30 mM of a fluorescently labeled glucose analog (2-NBDG, Thermo Fisher, Cat#N13195). Subsequently, the cells were washed twice with PBS and promptly analyzed by flow cytometry while maintaining the samples on ice.

Test by Glucose Uptake-Glo Assay (Promega, Cat#J1341), hypofunctional CAR-T cells were cultured for 4 days in the presence or absence of miltefosine (15 μM) or BAY-876 (1 μM). Afterward, the cells were washed with PBS and add 50 μL 1mM 2DG per well (3×10^5^ cells), shake briefly, and incubate 10 min at room temperature. Add 25 μL of Stop Buffer and shake briefly. Add 25 μL of Neutralization Buffer and shake briefly. Add 100 μL of 2DG6P Detection Reagent and shake briefly. Incubate for 1 h at room temperature. Then record luminescence using a 0.5 s integration on a luminometer.

#### *In vivo* mouse studies

Tumor xenograft model and *in vivo* CAR-T cells function detection was performed as previously described.^27^ Briefly, for NCI-H226 CDX mouse model, 6-8-week-old NPG mice were injected with 2×10^6^ NCI-H226-luciferase cells at right flank via subcutaneously injection in a volume of 50 μL DPBS and 50 μL Matrigel matrix (Corning, Cat#354277). Until Tumor size were approximately at 200-300mm^3^, the mice were randomly grouped. 2×10^6^ CAR-T cells were administrated intravenously twice with a one-week-interval. Miltefosine was administered intraperitoneally once a day at a dose of 10 mg/kg, and PD-1 antibody (Selleck, Cat#A2005) was administered intraperitoneally twice a week at a dose of 5 mg/kg. We recorded tumor twice a week. The tumor volume was calculated according to the following formula: tumor volume = (major axis of tumor) × (minor axis of tumor)^23^./2. Peripheral blood collected from orbital blood and the proportion of CAR-T cells was analyzed. In the end of experiment, tumor tissues were fixed and embedded in paraffin. The expression of Mesothelin (ZSGB-BIO, Cat#ZA-0579) and hCD3 (ZSGB-BIO, Cat#ZA-0503) in tumor sections were detected by Immunohistochemical staining.

For PDX mouse model, NPG mice engrafted with pancreatic carcinoma patient-derived xenograft on the right flank (PDX, Vitalstar). The group division, CAR-T cell injection, drug administration, recording of tumor size, analysis of the proportion of CAR-T cells in peripheral bloods and tumor were same as CDX model.

For B16F10-OVA murine tumor model, 8-10-week-old C57BL/6J mice were injected subcutaneously with 2 × 10^5^ B16F10-OVA cells on the right flank and randomly grouped. Miltefosine was administered intraperitoneally every other day at a dose of 10 mg/kg. On day 5, 1.5×10^6^ OT-1 cells were adoptively transferred via tail vein injection. Tumor size was monitored every two days.

### Quantification and statistical analysis

Data were analyzed using Prism 10 and are presented as mean ± standard error of the mean (SEM). Unpaired two-tailed Student’s t test was employed to assess differences between two experimental groups, with a *p*-value <0.05 deemed statistically significant. Sample sizes for animal studies were determined based on prior experiments. All experiments were conducted under blinded conditions, and the investigator analyzing the data was unaware of the identities of the control and treatment groups. Detailed statistical methods are outlined in the STAR Methods section or figure legends.

## Supplemental information

Document S1. Figures S1–S7 and Table S1

Document S2. Article plus supplemental information

## References

[1] Wherry E.J., Kurachi M. (2015). Molecular and cellular insights into T cell exhaustion. Nat. Rev. Immunol..

[2] Collier J.L., Weiss S.A., Pauken K.E., Sen D.R., Sharpe A.H. (2021). Not-so-opposite ends of the spectrum: CD8(+) T cell dysfunction across chronic infection, cancer and autoimmunity. Nat. Immunol..

[3] Wherry E.J. (2011). T cell exhaustion. Nat. Immunol..

[4] Wang Q., Qin Y., Li B. (2023). CD8(+) T cell exhaustion and cancer immunotherapy. Cancer Lett..

[5] Zajac A.J., Blattman J.N., Murali-Krishna K., Sourdive D.J., Suresh M., Altman J.D., Ahmed R. (1998). Viral immune evasion due to persistence of activated T cells without effector function. J. Exp. Med..

[6] Man K., Gabriel S.S., Liao Y., Gloury R., Preston S., Henstridge D.C., Pellegrini M., Zehn D., Berberich-Siebelt F., Febbraio M.A. (2017). Transcription Factor IRF4 Promotes CD8(+) T Cell Exhaustion and Limits the Development of Memory-like T Cells during Chronic Infection. Immunity.

[7] Bengsch B., Ohtani T., Khan O., Setty M., Manne S., O'Brien S., Gherardini P.F., Herati R.S., Huang A.C., Chang K.M. (2018). Epigenomic-Guided Mass Cytometry Profiling Reveals Disease-Specific Features of Exhausted CD8 T Cells. Immunity.

[8] McKinney E.F., Lee J.C., Jayne D.R.W., Lyons P.A., Smith K.G.C. (2015). T-cell exhaustion, co-stimulation and clinical outcome in autoimmunity and infection. Nature.

[9] Gruener N.H., Lechner F., Jung M.C., Diepolder H., Gerlach T., Lauer G., Walker B., Sullivan J., Phillips R., Pape G.R., Klenerman P. (2001). Sustained dysfunction of antiviral CD8+ T lymphocytes after infection with hepatitis C virus. J. Virol..

[10] Reignat S., Webster G.J.M., Brown D., Ogg G.S., King A., Seneviratne S.L., Dusheiko G., Williams R., Maini M.K., Bertoletti A. (2002). Escaping high viral load exhaustion: CD8 cells with altered tetramer binding in chronic hepatitis B virus infection. J. Exp. Med..

[11] Vardhana S.A., Hwee M.A., Berisa M., Wells D.K., Yost K.E., King B., Smith M., Herrera P.S., Chang H.Y., Satpathy A.T. (2020). Impaired mitochondrial oxidative phosphorylation limits the self-renewal of T cells exposed to persistent antigen. Nat. Immunol..

[12] Miller B.C., Sen D.R., Al Abosy R., Bi K., Virkud Y.V., LaFleur M.W., Yates K.B., Lako A., Felt K., Naik G.S. (2019). Subsets of exhausted CD8(+) T cells differentially mediate tumor control and respond to checkpoint blockade. Nat. Immunol..

[13] Im S.J., Hashimoto M., Gerner M.Y., Lee J., Kissick H.T., Burger M.C., Shan Q., Hale J.S., Lee J., Nasti T.H. (2016). Defining CD8+ T cells that provide the proliferative burst after PD-1 therapy. Nature.

[14] Philip M., Fairchild L., Sun L., Horste E.L., Camara S., Shakiba M., Scott A.C., Viale A., Lauer P., Merghoub T. (2017). Chromatin states define tumour-specific T cell dysfunction and reprogramming. Nature.

[15] Zheng C., Zheng L., Yoo J.K., Guo H., Zhang Y., Guo X., Kang B., Hu R., Huang J.Y., Zhang Q. (2017). Landscape of Infiltrating T Cells in Liver Cancer Revealed by Single-Cell Sequencing. Cell.

[16] Guo X., Zhang Y., Zheng L., Zheng C., Song J., Zhang Q., Kang B., Liu Z., Jin L., Xing R. (2018). Global characterization of T cells in non-small-cell lung cancer by single-cell sequencing. Nat. Med..

[17] Zhang L., Yu X., Zheng L., Zhang Y., Li Y., Fang Q., Gao R., Kang B., Zhang Q., Huang J.Y. (2018). Lineage tracking reveals dynamic relationships of T cells in colorectal cancer. Nature.

[18] Weber E.W., Parker K.R., Sotillo E., Lynn R.C., Anbunathan H., Lattin J., Good Z., Belk J.A., Daniel B., Klysz D. (2021). Transient rest restores functionality in exhausted CAR-T cells through epigenetic remodeling. Science.

[19] Ribas A., Wolchok J.D. (2018). Cancer immunotherapy using checkpoint blockade. Science.

[20] Alfei F., Kanev K., Hofmann M., Wu M., Ghoneim H.E., Roelli P., Utzschneider D.T., von Hoesslin M., Cullen J.G., Fan Y. (2019). TOX reinforces the phenotype and longevity of exhausted T cells in chronic viral infection. Nature.

[21] Khan O., Giles J.R., McDonald S., Manne S., Ngiow S.F., Patel K.P., Werner M.T., Huang A.C., Alexander K.A., Wu J.E. (2019). TOX transcriptionally and epigenetically programs CD8(+) T cell exhaustion. Nature.

[22] Seo H., Chen J., González-Avalos E., Samaniego-Castruita D., Das A., Wang Y.H., López-Moyado I.F., Georges R.O., Zhang W., Onodera A. (2019). TOX and TOX2 transcription factors cooperate with NR4A transcription factors to impose CD8(+) T cell exhaustion. Proc. Natl. Acad. Sci. USA.

[23] Scott A.C., Dündar F., Zumbo P., Chandran S.S., Klebanoff C.A., Shakiba M., Trivedi P., Menocal L., Appleby H., Camara S. (2019). TOX is a critical regulator of tumour-specific T cell differentiation. Nature.

[24] Yao C., Sun H.W., Lacey N.E., Ji Y., Moseman E.A., Shih H.Y., Heuston E.F., Kirby M., Anderson S., Cheng J. (2019). Single-cell RNA-seq reveals TOX as a key regulator of CD8(+) T cell persistence in chronic infection. Nat. Immunol..

[25] Chen J., López-Moyado I.F., Seo H., Lio C.W.J., Hempleman L.J., Sekiya T., Yoshimura A., Scott-Browne J.P., Rao A. (2019). NR4A transcription factors limit CAR T cell function in solid tumours. Nature.

[26] Liu X., Wang Y., Lu H., Li J., Yan X., Xiao M., Hao J., Alekseev A., Khong H., Chen T. (2019). Genome-wide analysis identifies NR4A1 as a key mediator of T cell dysfunction. Nature.

[27] Zhang X., Zhang C., Qiao M., Cheng C., Tang N., Lu S., Sun W., Xu B., Cao Y., Wei X. (2022). Depletion of BATF in CAR-T cells enhances antitumor activity by inducing resistance against exhaustion and formation of central memory cells. Cancer Cell.

[28] Quigley M., Pereyra F., Nilsson B., Porichis F., Fonseca C., Eichbaum Q., Julg B., Jesneck J.L., Brosnahan K., Imam S. (2010). Transcriptional analysis of HIV-specific CD8+ T cells shows that PD-1 inhibits T cell function by upregulating BATF. Nat. Med..

[29] Trefny M.P., Kirchhammer N., Auf der Maur P., Natoli M., Schmid D., Germann M., Fernandez Rodriguez L., Herzig P., Lötscher J., Akrami M. (2023). Deletion of SNX9 alleviates CD8 T cell exhaustion for effective cellular cancer immunotherapy. Nat. Commun..

[30] Kumar J., Kumar R., Kumar Singh A., Tsakem E.L., Kathania M., Riese M.J., Theiss A.L., Davila M.L., Venuprasad K. (2021). Deletion of Cbl-b inhibits CD8(+) T-cell exhaustion and promotes CAR T-cell function. J. Immunother. Cancer.

[31] Tang N., Cheng C., Zhang X., Qiao M., Li N., Mu W., Wei X.F., Han W., Wang H. (2020). TGF-beta inhibition via CRISPR promotes the long-term efficacy of CAR T cells against solid tumors. JCI Insight.

[32] Belk J.A., Yao W., Ly N., Freitas K.A., Chen Y.T., Shi Q., Valencia A.M., Shifrut E., Kale N., Yost K.E. (2022). Genome-wide CRISPR screens of T cell exhaustion identify chromatin remodeling factors that limit T cell persistence. Cancer Cell.

[33] Behrens G., Edelmann S.L., Raj T., Kronbeck N., Monecke T., Davydova E., Wong E.H., Kifinger L., Giesert F., Kirmaier M.E. (2021). Disrupting Roquin-1 interaction with Regnase-1 induces autoimmunity and enhances antitumor responses. Nat. Immunol..

[34] Wei J., Long L., Zheng W., Dhungana Y., Lim S.A., Guy C., Wang Y., Wang Y.D., Qian C., Xu B. (2019). Targeting REGNASE-1 programs long-lived effector T cells for cancer therapy. Nature.

[35] Mai D., Johnson O., Reff J., Fan T.J., Scholler J., Sheppard N.C., June C.H. (2023). Combined disruption of T cell inflammatory regulators Regnase-1 and Roquin-1 enhances antitumor activity of engineered human T cells. Proc. Natl. Acad. Sci. USA.

[36] Lynn R.C., Weber E.W., Sotillo E., Gennert D., Xu P., Good Z., Anbunathan H., Lattin J., Jones R., Tieu V. (2019). c-Jun overexpression in CAR T cells induces exhaustion resistance. Nature.

[37] Legut M., Gajic Z., Guarino M., Daniloski Z., Rahman J.A., Xue X., Lu C., Lu L., Mimitou E.P., Hao S. (2022). A genome-scale screen for synthetic drivers of T cell proliferation. Nature.

[38] Adachi K., Kano Y., Nagai T., Okuyama N., Sakoda Y., Tamada K. (2018). IL-7 and CCL19 expression in CAR-T cells improves immune cell infiltration and CAR-T cell survival in the tumor. Nat. Biotechnol..

[39] Hashimoto M., Kamphorst A.O., Im S.J., Kissick H.T., Pillai R.N., Ramalingam S.S., Araki K., Ahmed R. (2018). CD8 T Cell Exhaustion in Chronic Infection and Cancer: Opportunities for Interventions. Annu. Rev. Med..

[40] Schuster S.J., Bishop M.R., Tam C.S., Waller E.K., Borchmann P., McGuirk J.P., Jäger U., Jaglowski S., Andreadis C., Westin J.R. (2019). Tisagenlecleucel in Adult Relapsed or Refractory Diffuse Large B-Cell Lymphoma. N. Engl. J. Med..

[41] Kuhlencord A., Maniera T., Eibl H., Unger C. (1992). Hexadecylphosphocholine: oral treatment of visceral leishmaniasis in mice. Antimicrob. Agents Chemother..

[42] Scott-Browne J.P., López-Moyado I.F., Trifari S., Wong V., Chavez L., Rao A., Pereira R.M. (2016). Dynamic Changes in Chromatin Accessibility Occur in CD8(+) T Cells Responding to Viral Infection. Immunity.

[43] Bengsch B., Johnson A.L., Kurachi M., Odorizzi P.M., Pauken K.E., Attanasio J., Stelekati E., McLane L.M., Paley M.A., Delgoffe G.M., Wherry E.J. (2016). Bioenergetic Insufficiencies Due to Metabolic Alterations Regulated by the Inhibitory Receptor PD-1 Are an Early Driver of CD8(+) T Cell Exhaustion. Immunity.

[44] Guo Y., Xie Y.Q., Gao M., Zhao Y., Franco F., Wenes M., Siddiqui I., Bevilacqua A., Wang H., Yang H. (2021). Metabolic reprogramming of terminally exhausted CD8(+) T cells by IL-10 enhances anti-tumor immunity. Nat. Immunol..

[45] Eibl H., Unger C. (1990). Hexadecylphosphocholine: a new and selective antitumor drug. Cancer Treat Rev..

[46] Wang D., Aguilar B., Starr R., Alizadeh D., Brito A., Sarkissian A., Ostberg J.R., Forman S.J., Brown C.E. (2018). Glioblastoma-targeted CD4+ CAR T cells mediate superior antitumor activity. JCI Insight.

[47] Good C.R., Aznar M.A., Kuramitsu S., Samareh P., Agarwal S., Donahue G., Ishiyama K., Wellhausen N., Rennels A.K., Ma Y. (2021). An NK-like CAR T cell transition in CAR T cell dysfunction. Cell.

[48] Gemta L.F., Siska P.J., Nelson M.E., Gao X., Liu X., Locasale J.W., Yagita H., Slingluff C.L., Hoehn K.L., Rathmell J.C., Bullock T.N.J. (2019). Impaired enolase 1 glycolytic activity restrains effector functions of tumor-infiltrating CD8(+) T cells. Sci. Immunol..

[49] Sinclair L.V., Barthelemy C., Cantrell D.A. (2020). Single cell glucose uptake assays: a cautionary tale. Immunometabolism.

[50] Rathmell J.C., Vander Heiden M.G., Harris M.H., Frauwirth K.A., Thompson C.B. (2000). In the absence of extrinsic signals, nutrient utilization by lymphocytes is insufficient to maintain either cell size or viability. Mol. Cell.

[51] Macintyre A.N., Gerriets V.A., Nichols A.G., Michalek R.D., Rudolph M.C., Deoliveira D., Anderson S.M., Abel E.D., Chen B.J., Hale L.P., Rathmell J.C. (2014). The glucose transporter Glut1 is selectively essential for CD4 T cell activation and effector function. Cell Metab..

[52] Palmer C.S., Ostrowski M., Gouillou M., Tsai L., Yu D., Zhou J., Henstridge D.C., Maisa A., Hearps A.C., Lewin S.R. (2014). Increased glucose metabolic activity is associated with CD4+ T-cell activation and depletion during chronic HIV infection. AIDS (London, England).

[53] Poorebrahim M., Melief J., Pico de Coaña Y., L Wickström S., Cid-Arregui A., Kiessling R. (2021). Counteracting CAR T cell dysfunction. Oncogene.

[54] Kunkele A., Johnson A.J., Rolczynski L.S., Chang C.A., Hoglund V., Kelly-Spratt K.S., Jensen M.C. (2015). Functional Tuning of CARs Reveals Signaling Threshold above Which CD8+ CTL Antitumor Potency Is Attenuated due to Cell Fas-FasL-Dependent AICD. Cancer Immunol. Res..

[55] Roselli E., Boucher J.C., Li G., Kotani H., Spitler K., Reid K., Cervantes E.V., Bulliard Y., Tu N., Lee S.B. (2021). 4-1BB and optimized CD28 co-stimulation enhances function of human mono-specific and bi-specific third-generation CAR T cells. J. Immunother. Cancer.

[56] Smith E.L., Staehr M., Masakayan R., Tatake I.J., Purdon T.J., Wang X., Wang P., Liu H., Xu Y., Garrett-Thomson S.C. (2018). Development and Evaluation of an Optimal Human Single-Chain Variable Fragment-Derived BCMA-Targeted CAR T Cell Vector. Mol. Ther..

[57] Dai Z., Mu W., Zhao Y., Cheng J., Lin H., Ouyang K., Jia X., Liu J., Wei Q., Wang M. (2022). T cells expressing CD5/CD7 bispecific chimeric antigen receptors with fully human heavy-chain-only domains mitigate tumor antigen escape. Signal Transduct. Target. Ther..

[58] Suematsu M., Yagyu S., Nagao N., Kubota S., Shimizu Y., Tanaka M., Nakazawa Y., Imamura T. (2022). PiggyBac Transposon-Mediated CD19 Chimeric Antigen Receptor-T Cells Derived From CD45RA-Positive Peripheral Blood Mononuclear Cells Possess Potent and Sustained Antileukemic Function. Front. Immunol..

[59] Heczey A., Louis C.U., Savoldo B., Dakhova O., Durett A., Grilley B., Liu H., Wu M.F., Mei Z., Gee A. (2017). CAR T Cells Administered in Combination with Lymphodepletion and PD-1 Inhibition to Patients with Neuroblastoma. Mol. Ther..

[60] Adusumilli P.S., Zauderer M.G., Rivière I., Solomon S.B., Rusch V.W., O'Cearbhaill R.E., Zhu A., Cheema W., Chintala N.K., Halton E. (2021). A Phase I Trial of Regional Mesothelin-Targeted CAR T-cell Therapy in Patients with Malignant Pleural Disease, in Combination with the Anti-PD-1 Agent Pembrolizumab. Cancer Discov..

[61] Bagchi S., Yuan R., Engleman E.G. (2021). Immune Checkpoint Inhibitors for the Treatment of Cancer: Clinical Impact and Mechanisms of Response and Resistance. Annu. Rev. Pathol..

[62] Beck H., Härter M., Haß B., Schmeck C., Baerfacker L. (2022). Small molecules and their impact in drug discovery: A perspective on the occasion of the 125th anniversary of the Bayer Chemical Research Laboratory. Drug Discov. Today.

[63] Dufva O., Koski J., Maliniemi P., Ianevski A., Klievink J., Leitner J., Pölönen P., Hohtari H., Saeed K., Hannunen T. (2020). Integrated drug profiling and CRISPR screening identify essential pathways for CAR T-cell cytotoxicity. Blood.

[64] Marro B.S., Zak J., Zavareh R.B., Teijaro J.R., Lairson L.L., Oldstone M.B.A. (2019). Discovery of Small Molecules for the Reversal of T Cell Exhaustion. Cell Rep..

[65] Martinez D.Y., Bravo-Cossio F., Valdivia-Tapia M.D.C., Carreazo N.Y., Cabello-Vilchez A.M. (2022). Successful Treatment of Primary Amoebic Meningoencephalitis Using a Novel Therapeutic Regimen Including Miltefosine and Voriconazole. Acta Parasitol..

[66] Verweij J., Gandia D., Planting A.S., Stoter G., Armand J.P. (1993). Phase II study of oral miltefosine in patients with squamous cell head and neck cancer. Eur. J. Cancer.

[67] Verweij J., Krzemieniecki K., Kok T., Poveda A., van Pottelsberghe C., van Glabbeke M., Mouridsen H. (1993). Phase II study of miltefosine (hexadecylphosphocholine) in advanced soft tissue sarcomas of the adult--an EORTC Soft Tissue and Bone Sarcoma Group Study. Eur. J. Cancer.

[68] Giroux Leprieur E., Dumenil C., Julie C., Giraud V., Dumoulin J., Labrune S., Chinet T. (2017). Immunotherapy revolutionises non-small-cell lung cancer therapy: Results, perspectives and new challenges. Eur. J. Cancer.

[69] O'Neill L.A.J., Kishton R.J., Rathmell J. (2016). A guide to immunometabolism for immunologists. Nat. Rev. Immunol..

[70] Uhl F.M., Chen S., O'Sullivan D., Edwards-Hicks J., Richter G., Haring E., Andrieux G., Halbach S., Apostolova P., Buscher J. (2020). Metabolic reprogramming of donor T cells enhances graft-versus-leukemia effects in mice and humans. Sci. Transl. Med..

[71] Shi Y., Kotchetkov I.S., Dobrin A., Hanina S.A., Rajasekhar V.K., Healey J.H., Sadelain M. (2024). GLUT1 overexpression enhances CAR T cell metabolic fitness and anti-tumor efficacy. Mol. Ther..

[72] Sun R.-x., Liu Y.-f., Sun Y.-s., Zhou M., Wang Y., Shi B.-z., Jiang H., Li Z.-h. (2024). GPC3-targeted CAR-T cells expressing GLUT1 or AGK exhibit enhanced antitumor activity against hepatocellular carcinoma. Acta Pharmacol. Sin..

[73] Zur R.T., Atar O., Barliya T., Hoogi S., Abramovich I., Gottlieb E., Ron-Harel N., Cohen C.J. (2024). Genetically engineering glycolysis in T cells increases their antitumor function. J. Immunother. Cancer.

[74] Muheeb B., Nazish A., Shazna T.F., Altorki N.K., McGraw T.E. (2017). Distinct Akt phosphorylation states are required for insulin regulated Glut4 and Glut1-mediated glucose uptake. Elife.

[75] Ren J., Liu X., Fang C., Jiang S., June C.H., Zhao Y. (2017). Multiplex Genome Editing to Generate Universal CAR T Cells Resistant to PD1 Inhibition. Clin. Cancer Res..

