# Supplementary material for: Miltefosine reinvigorates exhausted T cells by targeting their bioenergetic state
Source: Cell Rep Med. 2024 Dec 9;5(12):101869. doi: 10.1016/j.xcrm.2024.101869 (PMC11722131; doi:10.1016/j.xcrm.2024.101869)
Supplement: Document S1. Figures S1–S7 and Table S1 [file mmc1.pdf]

Cell Reports Medicine, Volume *5*

Supplemental information

Miltefosine reinvigorates exhausted T cells

by targeting their bioenergetic state

Xingying Zhang, Chenze Zhang, Shan Lu, Jingxi Dong, Na Tang, Yao Wang, Weidong Han, Xi Pan, Xiang Zhang, Duan Liu, Ng Shyh-Chang, Yu Wang, Guihai Feng, and Haoyi Wang

Supplemental Figures

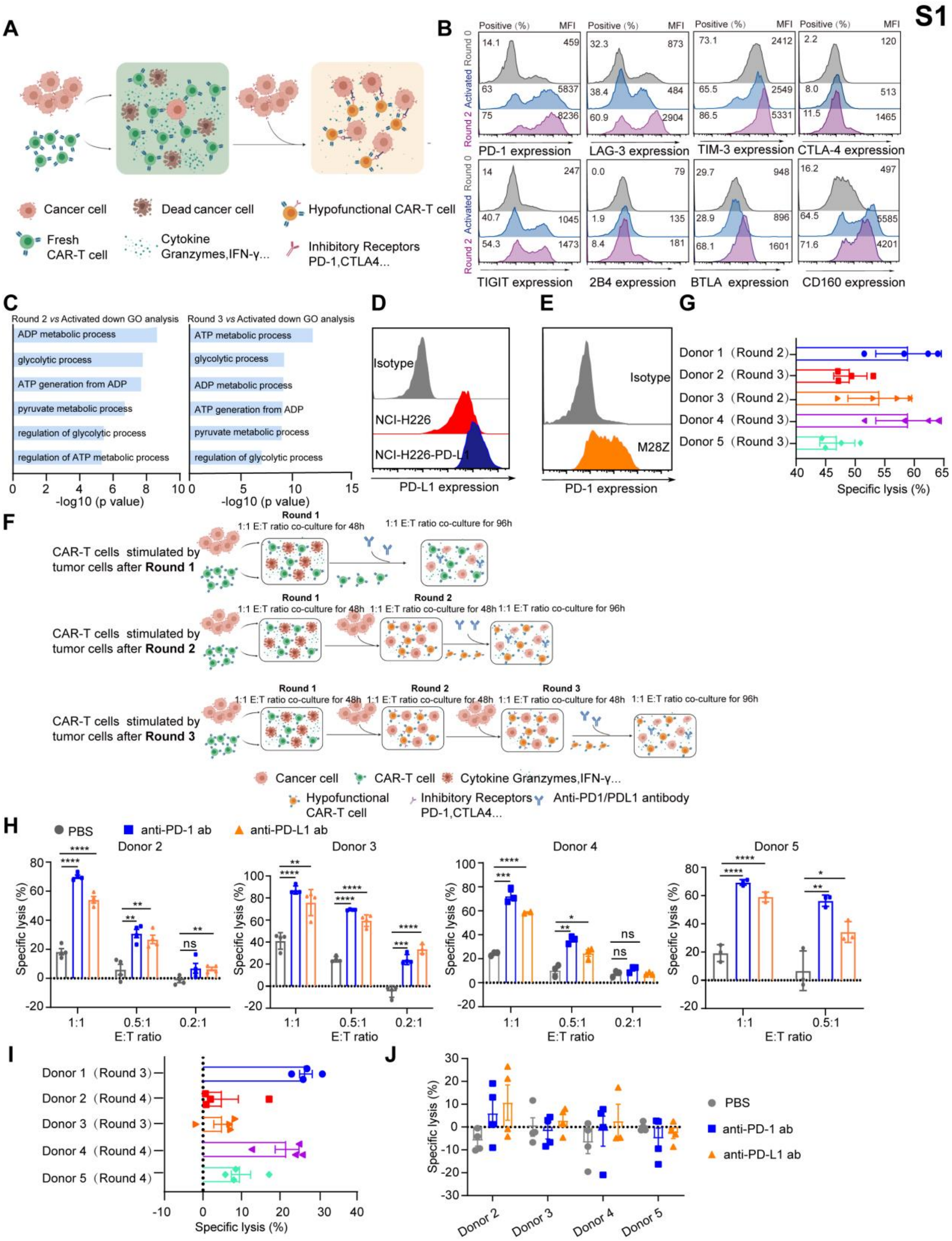

Figure S1: Hypofunctional CAR-T cells display exhaustion hallmarks and can be rescued by anti-PD-1/PD-L1 antibody (related to Figure 1).

(A) The schematic diagram of CAR-T cell hypofunction model *in vitro*.

(B) The cell surface expression of exhaustion-related makers on CAR-T cells.

(C) GO analysis was conducted on the downregulated genes of Round 3 and Round 2 M28Z cells, as

compared with activated M28Z cells.

(D) The cell surface expression of PD-L1 on NCI-H226 and NCI-H226-PD-L1 cells.

(E) The cell surface expression of PD-1 on M28Z CAR-T cells.

(F) The schematic diagram illustrates the specific lysis of NCI-H226-luciferase cells after coculture with round 1, round 2 and round 3 M28Z CAR-T cells (Donor 1) with anti-PD-1 or anti-PD-L1 antibody treatment for 4 days at a 1:1 E:T ratio (related to Figure 1G)

(G) The specific lysis of NCI-H226-luciferase after co-culture with different donors derived CAR-T cells (n=4, related to Figure 1A).

(H) The specific lysis of NCI-H226-luciferase after co-culture with hypofunctional M28Z (Donors 2-5) at different E:T ratios for 4 days with anti-PD-1 or PD-L1 antibody treatment (n=4, related to Figure 1H).

(I) The specific lysis of NCI-H226-luciferase after co-culture with different donors derived CAR-T cells (n=4, related to Figure 1A).

(J) The specific lysis of NCI-H226-luciferase after co-culture with hypofunctional M28Z in (Donors 2-5) at different E:T ratios for 4 days with anti-PD-1 or PD-L1 antibody treatment (n=4).

*Unpaired t test was used in statistical analysis. NS, not significant, *p<0.05, **p<0.01, ***p<0.001, ****p<0.0001. All error bars denote SEM.*

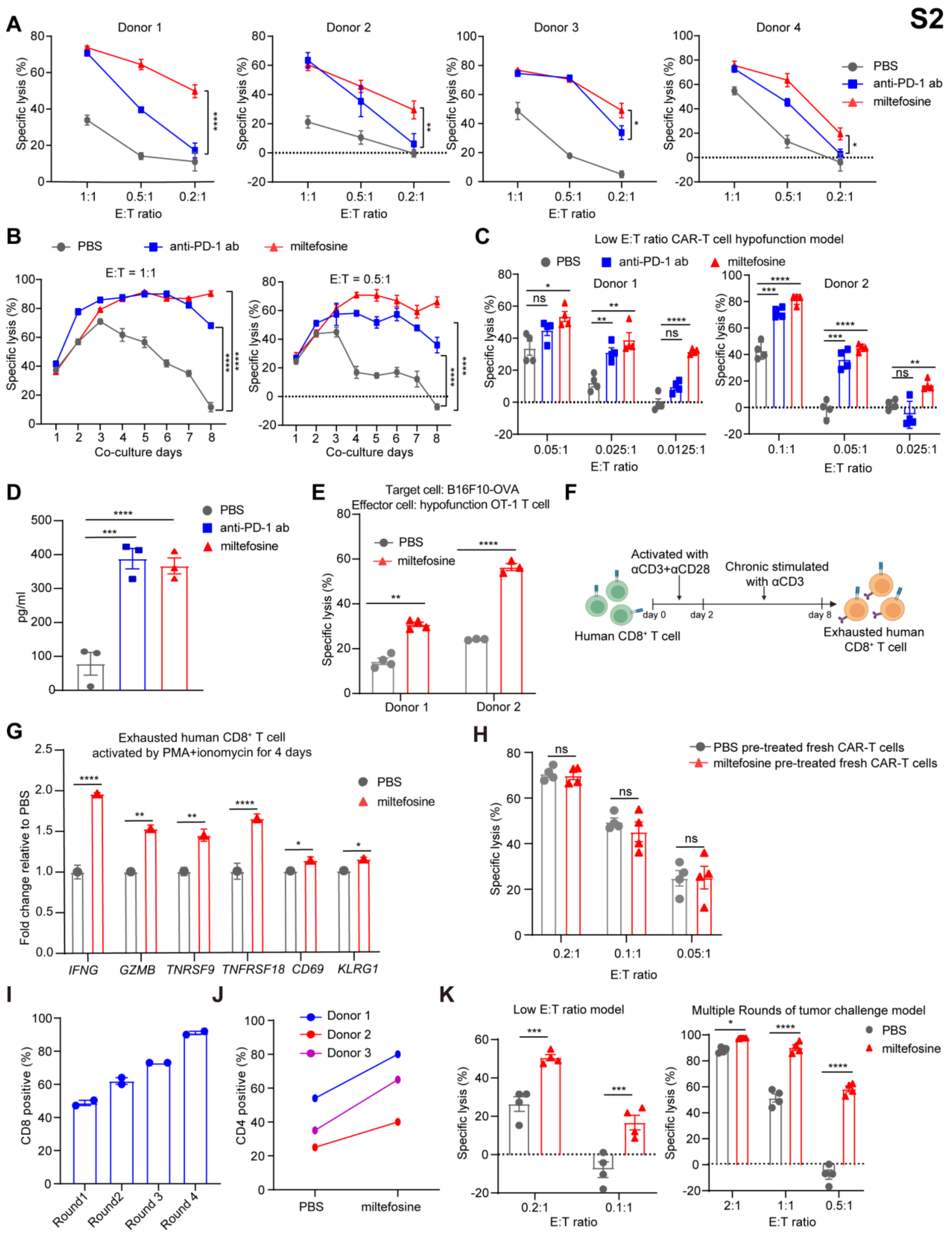

Figure S2：Miltefosine can enhance the antitumor efficacy of hypofunctional CAR-T and T cells (related to Figure 2).

(A) The specific lysis of NCI-H226-luciferase after co-culture with progenitor exhausted-like M28Z CAR-T cells with miltefosine or anti-PD-1 antibody treatment (n=4, related to Figure 2J).

(B) Time-dependent specific lysis of NCI-H226-luciferase after co-culture with progenitor-like hypofunctional M28Z at 1:1 and 0.5:1 E:T ratio with miltefosine or anti-PD-1 antibody treatment

(n=4).

(C) The specific lysis of NCI-H226-luciferase after co-culture with fresh M28Z with miltefosine or anti-PD-1 antibody treatment at different low E:T ratios for 4 days (n=4).

(D) The secretion of IFNγ by hypofunctional M28Z cells after coculture with NCI-H226-luciferase cells treated with miltefosine or anti-PD-1 antibody at 1:1 effector-to-target (E: T) ratio for 4 days (n=3).

(E) The specific tumor killing of hypofunctional OT-1 cells from two donors, with PBS or miltefosine treatment at a 1:1 E:T ratio for 4 days (n=3).

(F) Schematic diagram of human T cell exhaustion inducing condition.

(G) Activation-related genes expression was analyzed by quantitative real-time PCR in exhausted human T cells treated with PBS or miltefosine after PMA (50 ng/ml) and ionomycin (1 μg/ml) stimulation (n=4).

(H) Fresh M28Z were pretreated with miltefosine or PBS for 4 days, and then cocultured with NCI-H226-luciferase cells at different E:T ratios (n=4).

(I) Percentage of CD8 positive CAR-T cells during multiple rounds of tumor challenge (n=2).

(J) Percentage of CD4 positive hypofunctional CAR-T cells treated with PBS or miltefosine for 4 days (n=3).

(K) The specific lysis of NCI-H226-luciferase after co-culture with $CD4^+$ CAR-T cells treated with PBS or miltefosine using a low E:T ratio CAR-T cell hypofunction model and multiple rounds of tumor challenge CAR-T cell hypofunction model (n=4).

*Unpaired t test was used in statistical analysis. NS, not significant, *p<0.05, **p<0.01, ***p<0.001, ****p<0.0001. All error bars denote SEM.*

S3

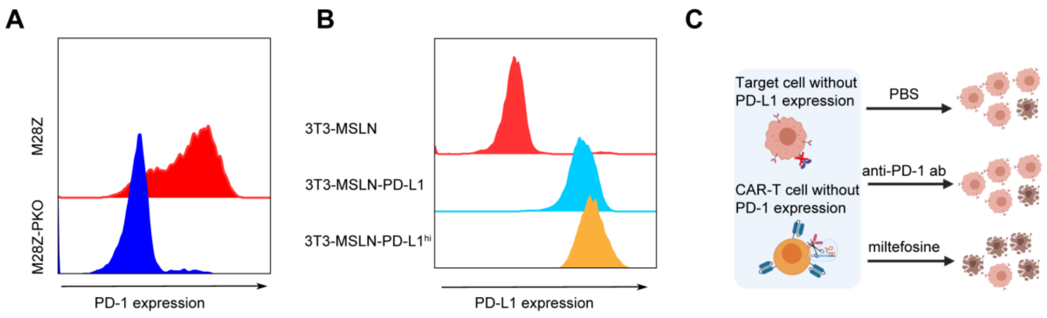

Figure S3:　The mechanism of miltefosine on hypofunctional CAR-T cell efficacy differs from anti-PD-1 antibody (related to Figure 3).

(A) The cell surface expression of PD-1 on M28Z and M28Z-PKO. M28Z-PKO, M28Z CAR-T cells with *PDCD1* knockout.

(B) The cell surface expression of PD-L1 on 3T3-MSLN, 3T3-MSLN-PD-L1 and 3T3-MSLN-PD-L1^{hi} cells. MSLN, Mesothelin.

(C) The schematic diagram of miltefosine's effect on hypofunctional CAR-T cells.

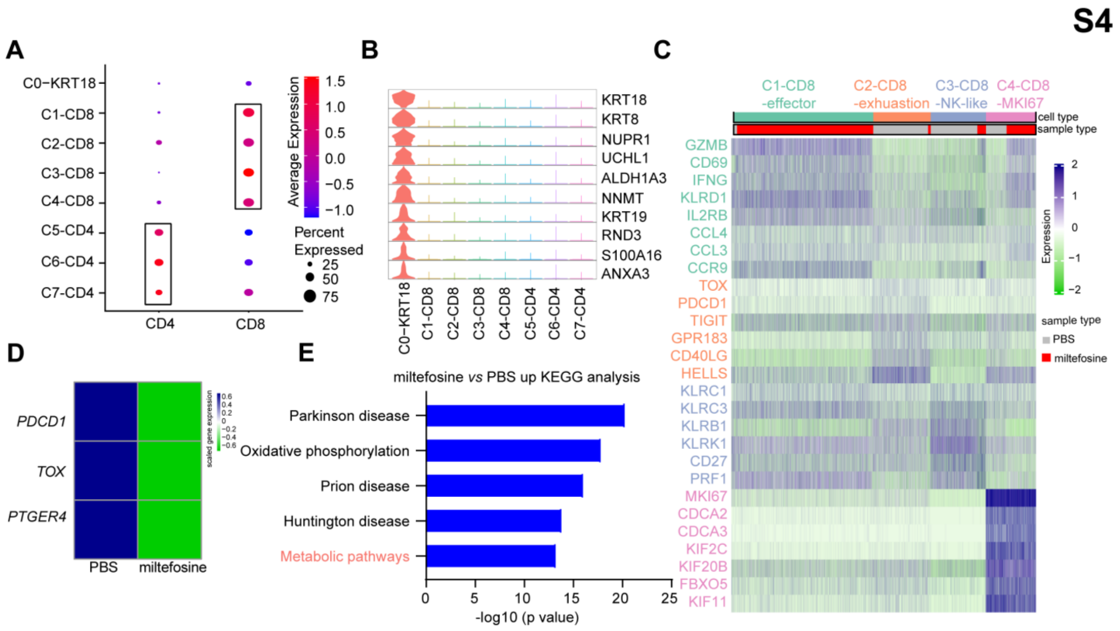

Figure S4: ScRNA-seq analysis of miltefosine's effect on hypofunctional CAR-T cells (related to Figure 4).

(A) Dot plot illustrating the expression level of CD4 and CD8 in each cluster.

(B) Dot plot illustrating the expression level of tumor-related genes in each cluster.

(C) Heatmap of maker genes for CD8^{+} cluster defined in Figure 4B (related to Figure 4D).

(D) Dot plot illustrating the expression level of activated- and exhaustion-related genes in PBS and miltefosine group.

(E) KEGG enrichment analysis across miltefosine and PBS group.

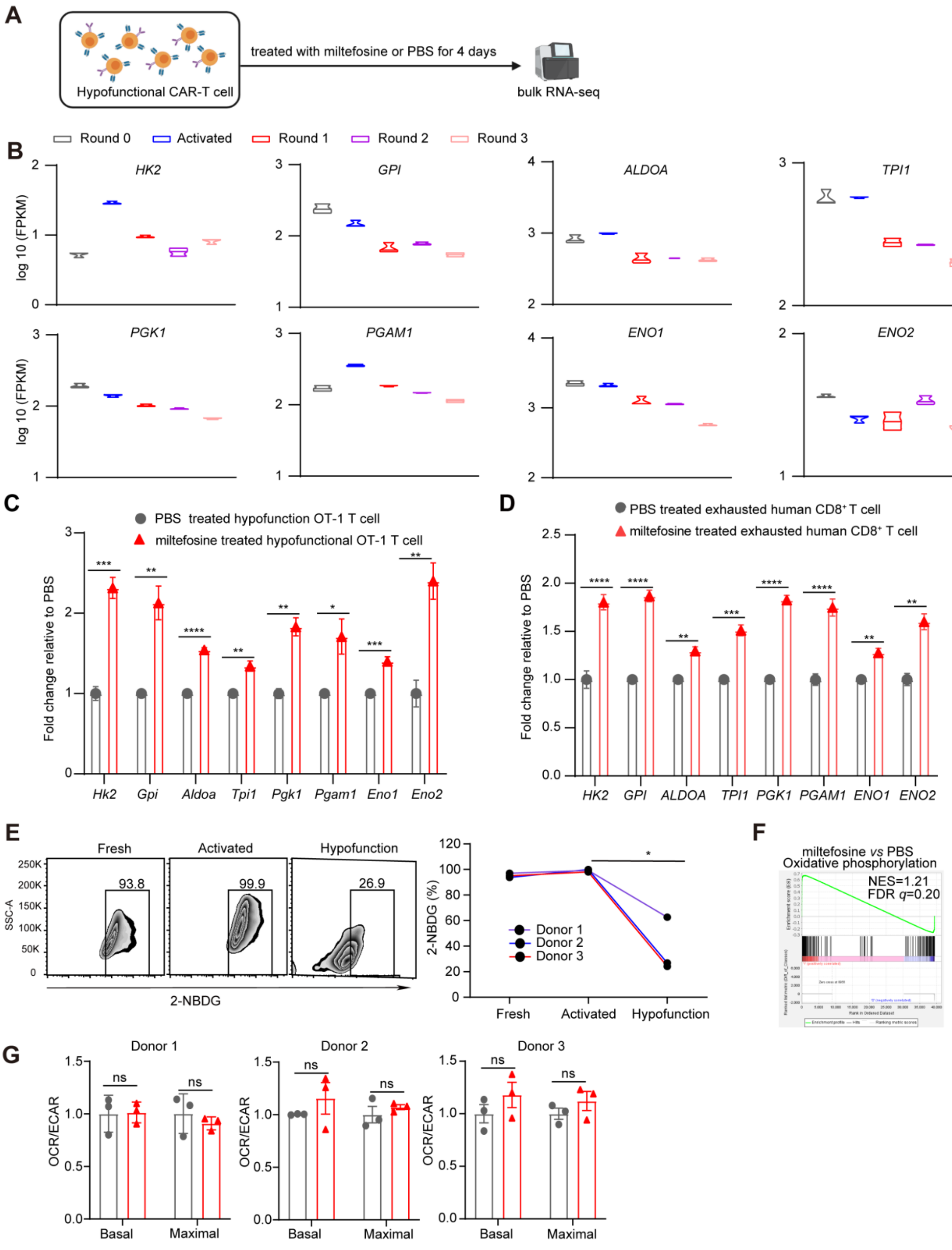

Figure S5: Miltefosine enhances the glycolytic function and glucose uptake of hypofunctional CAR-T and T cells (related to Figure 5).

(A) The schematic diagram of sample preparation for bulk RNA- sequencing.

(B) The expression of glycolysis-related genes in CAR-T cells during multiple tumor challenges (n=3).

(C) The expression of glycolysis-related genes was analyzed by quantitative real-time PCR in hypofunctional OT-1 T cells treated with PBS or miltefosine (n=4).

(D) The expression of glycolysis-related genes was analyzed by quantitative real-time PCR in exhausted human T cells treated with miltefosine for 4 days (n=4).

(E) The 2-NBDG uptake in fresh, activated and hypofunctional CAR-T cells derived from three independent donors.

(F) GSEA shown oxidative phosphorylation related genes were enriched in miltefosine treated hypofunctional CAR-T cells.

(G) Ratios of basal and maximal OCR to ECAR in hypofunctional CAR-T cells treated with miltefosine, derived from three different donors (n=3).

Unpaired t test was used in statistical analysis. *NS, not significant*, *p<0.05, **p<0.*01, ***p<0.001, ****p<0.0001. All error bars denote SEM.*

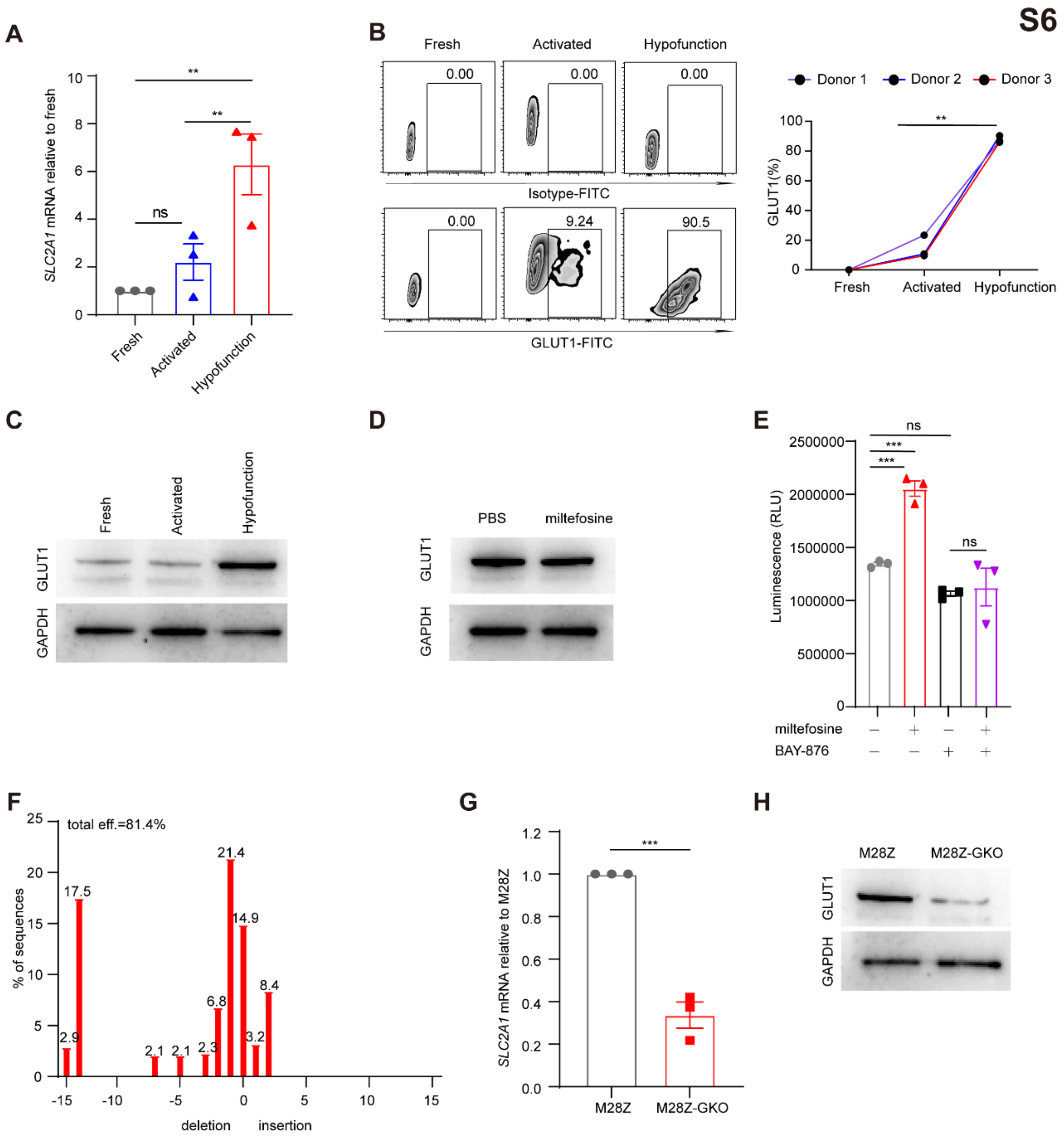

Figure S6：Miltefosine restores the impaired glucose uptake of hypofunctional CAR-T cells through GLUT1 (related to Figure 6).

(A) The expression of *SLC2A1* (encoding GLUT1) in fresh, activated and hypofunctional CAR-T cells evaluated by quantitative real-time PCR (n=3).

(B) The expression of GLUT1 in fresh, activated and hypofunctional CAR-T cells derived from three independent donors (n=3).

(C) The expression of GLUT1 in fresh, activated and hypofunctional CAR-T cells evaluated by western blot.

(D) The expression of GLUT1 in hypofunctional CAR-T cells treated with PBS or miltefosine evaluated by western blot.

(E) Glucose uptake in hypofunctional CAR-T cells treated with PBS or miltefosine combined with BAY-876, measured using the Glucose Uptake-Glo™ Assay (n=3). The donor referred to as "donor 1" in Figure 5G was also used in this figure.

(F) The GLUT1 editing efficiency of M28Z-GKO CAR-T cells evaluated by TIDE.

(G) The GLUT1 editing efficiency of M28Z-GKO CAR-T cells evaluated by quantitative real-time PCR (n=3).

(H) GLUT1 editing efficiency of M28Z-GKO CAR-T cells evaluated by western blot.

*Unpaired t test was used in statistical analysis. NS, not significant, **p<0.01, ***p<0.001. All error bars denote SEM.*

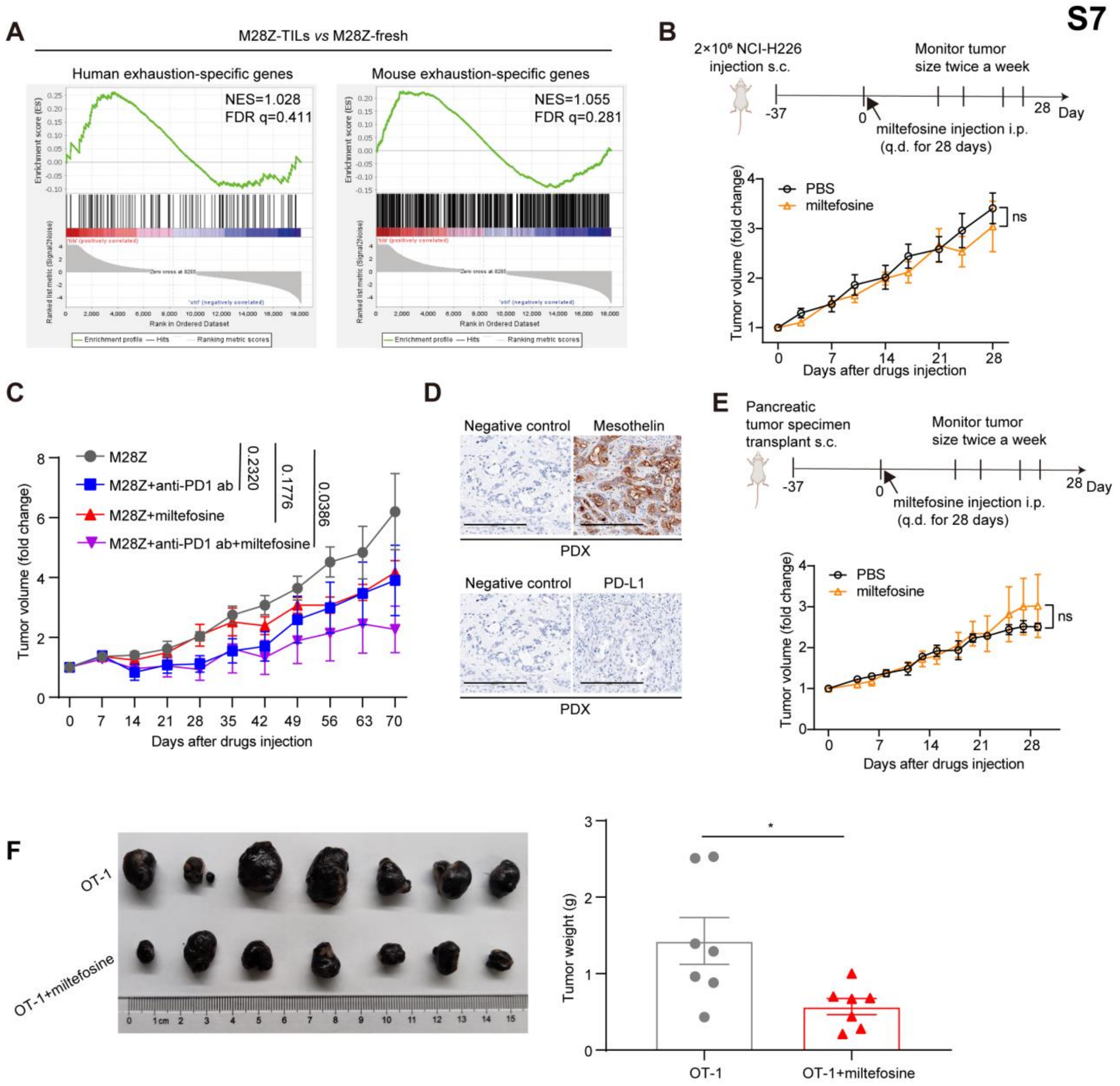

Figure S7: Miltefosine improves the anti-tumor efficacy of T cells *in vivo* (related to Figure 7).

(A) GSEA of genes up-regulated in $CD8^+$ exhausted T cells in liver cancer, colorectal cancer and non-small-cell lung cancer, and up-regulated genes in $CD8^+$ exhaustion T cells compared with effector $CD8^+$ T cells in chronic LCMV infection. NES: normalized enrichment score. Genes, from the left to right of the rank-ordered were enriched in M28Z-TILs and M28Z-fresh group, respectively. The analysis method is consistent with that described in Figure 1D.

(A) The effect of miltefosine on tumor growth in CDX model (n=4).

(B) The fold change of tumor volume in CAR-T cell treated CDX mouse model in the presence or absence of miltefosine and anti-PD-1 antibody administration (n=4).

(C) Immunohistochemical staining showed the expression of mesothelin and PD-L1 in the PDX tumor sample. Scale bar: 200 μm.

(D) The effect of miltefosine on tumor growth in PDX model (n=4).

(E) Tumor photo and tumor weight of each group at the end of experiment (n=7).

*Unpaired t test was used in statistical analysis. NS, not significant, *$p<0.05$. All error bars denote SEM.*

Table S1: Primers for quantitative real-time PCR

Gene name	Sequence
GAPDH	F: GTCTCCTCTGACTTCAACAGCG<br>R: ACCACCCTGTTGCTGTAGCCAA
IFNG	F: GAGTGTGGAGACCATCAAGGAAG<br>R: TGCTTTGCGTTGGACATTCAAGTC
GZMB	F: CGACAGTACCATTGAGTTGTGCG<br>R: TTCGTCCATAGGAGACAATGCCC
TNFRSF9	F: TCTTCCTCACGCTCCGTTTCTC<br>R: TGGAAATCGGCAGCTACAGCCA
TNFRSF18	F: CCAGTGTATCGACTGTGCCTCG<br>R: CACAGCGTTGTGGGTCTTGTTC
CD69	F: GCTGGACTTCAGCCCAAAATGC<br>R: AGTCCAACCCAGTGTTCCTCTC
KLRG1	F: CCTTTTGCTGGATTGGTCTGAGG<br>R: TTGATGGCACCGCATGTCTGCA
HK2	F: GAGTTTGACCTGGATGTGGTTGC<br>R: CCTCCATGTAGCAGGCATTGCT
GPI	F: CTGGTAGACGGCAAGGATGTGA<br>R: TCCGTGATGGTCTTGCCTGTGT
ALDOA	F: GACACTCTACCAGAAGGCGGAT<br>R: GGTGGTAGTCTCGCCATTTGTC
TPI1	F: CGAGCAGACAAAGGTCATCGCA<br>R: TCGGAGCTTCTCGTGTACTTCC
PGK1	F: CCGCTTTCATGTGGAGGAAGAAG<br>R: CTCTGTGAGCAGTGCCAAAAGC
PGAM1	F: GCTCTGCCCTTCTGGAATGAAG<br>R: ATACCAGTCGGCAGGTTCAGCT
ENO1	F: AGTCAACCAGATTGGCTCCGTG<br>R: CACAACCAGGTCAGCGATGAAG
ENO2	F: CTGTATCGCCACATTGCTCAGC<br>R: AGCTTGTTGCCAGCATGAGAGC
SLC2A1	F: CATGCAGTGAATCTGTGGCA<br>R: GGCTGGGTCAGAGGTAATAC

Gapdh	F: CATCACTGCCACCCAGAAGACTG<br>R: ATGCCAGTGAGCTTCCCGTTCAG
Hk2	F: CCCTGTGAAGATGTTGCCCACT<br>R: CCTTCGCTTGCCATTACGCACG
Gpi	F: GAACCCGCAGTTCCAGAAGC<br>R: TGATGCAGATGCTGGTGGAG
Aldoa	F: CACGAGACACTGTACCAGAAGG<br>R: TTGTCTCGCCATTGGTTCCTGC
Tpi1	F: GGCAACTGGAAGATGAACGGGA<br>R: CTGGCAAAGTCGATGTAAGCGG
Pgk1	F: GATGCTTTCCGAGCCTCACTGT<br>R: ACCAGCCTTCTGTGGCAGATTC
Pgam1	F: CCCCTTCTACAGCAACATCAGC<br>R: GCTCTGGCAATAGTGTCCTTCAG
Eno1	F: TACCGCCACATTGCTGACTTGG<br>R: GCTTGTTGCCAGCATGAGAACC
Eno2	F: TGGCAAGGATGCCACTAACGTG<br>R: AACTCAGAGGCAGCCACATCCA
